# Gender and geographical bias in the editorial decision-making process of biomedical journals: a case-control study

**DOI:** 10.1136/bmjebm-2024-113083

**Published:** 2024-12-25

**Authors:** Angèle Gayet-Ageron, Khaoula Ben Messaoud, Mark Richards, Cyril Jaksic, Julien Gobeill, Jeevanthi Liyanapathirana, Luc Mottin, Nona Naderi, Patrick Ruch, Zoé Mariot, Alexandra Calmy, Julia Friedman, Leonard Leibovici, Sara Schroter

**Affiliations:** 1 Division of Clinical Epidemiology, University Hospitals Geneva, Geneva, Switzerland; 2 Department of Health and Community Medicine, University of Geneva, Geneva, Switzerland; 3 BMJ Publishing Group, London, UK; 4 SIB Text Mining, Swiss Institute of Bioinformatics Geneva, Geneva, Switzerland; 5 BiTeM Group, Information Sciences, HES-SO Geneve, Le Lignon, Switzerland; 6 CNRS, Laboratoire Interdisciplinaire des Sciences du Numérique, Université Paris-Saclay, Gif-sur-Yvette, Île-de-France, France; 7 Internal Medicine Division, Hôpital Riviera-Chablais, Rennaz, Switzerland; 8 Division of Infectious Diseases, HIV Unit, Geneva University Hospitals, Geneva, Switzerland; 9 Clinical Microbiology & Infection Editorial Office, Rabin Medical Center, Petah Tikva, Israel; 10 Internal Medicine E, Rabin Medical Center, Petah Tikva, Israel; 11 Faculty of Medicine, Tel Aviv University, Tel Aviv, Israel; 12 Faculty of Public Health and Policy, London School of Hygiene and Tropical Medicine, London, UK

**Keywords:** Leadership, Methods, Publishing, Sociology, Medical

## Abstract

**Objectives:**

To assess whether the gender (primary) and geographical affiliation (post-hoc) of the first and/or last authors are associated with publication decisions after peer review.

**Design:**

Case-control study.

**Setting:**

Biomedical journals.

**Participants:**

Original peer-reviewed manuscripts submitted between 1 January 2012 and 31 December 2019.

**Main outcome measure:**

Manuscripts accepted (cases) and rejected for publication (controls).

**Results:**

Of 6213 included manuscripts, 5294 (85.2%) first and 5479 (88.1%) last authors’ gender were identified; 2511 (47.4%) and 1793 (32.7%) were women, respectively. The proportion of women first and last authors was 48.4% (n=1314) and 32.2% (n=885) among cases and 46.4% (n=1197) and 33.2% (n=908) among controls. After adjustment, the association between the first author’s gender and acceptance for publication remained non-significant 1.04 (0.92 to 1.17). Acceptance for publication was lower for first authors affiliated to Asia 0.58 (0.46 to 0.73), Africa 0.75 (0.41 to 1.36) and South America 0.68 (0.40 to 1.16) compared with Europe, and for first author affiliated to upper-middle country-income 0.66 (0.47 to 0.95) and lower-middle/low 0.69 (0.46 to 1.03) compared with high country-income group. It was significantly higher when both first and last authors were affiliated to different countries from same geographical and income groups 1.35 (1.03 to 1.77), different countries and geographical but same income groups 1.50 (1.14 to 1.96) or different countries, geographical and income groups 1.78 (1.27 to 2.50) compared with authors from similar countries. The study funding was independently associated with the acceptance for publication (when compared with no funding, 1.40; 1.04 to 1.89 for funding by association & foundations, 2.76; 1.87 to 4.10 for international organisations, 1.30; 1.04 to 1.62 for non-profit & associations & foundations). The reviewers’ recommendations of the original submitted version were significantly associated with the outcome (unadjusted 5.36; 4.98 to 5.78 for acceptance compared with rejection). Gender of the first author was not associated with reviewers’ recommendations (adjusted 0.96, 0.87 to 1.06).

**Conclusions:**

We did not identify evidence of gender bias during the editorial decision-making process for papers sent out to peer review. However, the under-representation in manuscripts accepted for publication of first authors affiliated to Asia, Africa or South America and those affiliated to upper/lower-middle and low country-income group, indicates poor representation of global scientists’ opinion and supports growing demands for improving equity, diversity and inclusion in biomedical research. The more diverse the countries and incomes of the first and last authors, the greater the chances of the publication being accepted.

**WHAT IS ALREADY KNOWN ON THIS SUBJECT**

- Published studies have revealed gender inequalities in academia, attainment of leadership positions, successful grant funding and in biomedical research production (including reported research contributions, attainment of key authorship positions in submitted and published manuscripts, membership of editorial boards, and participation in peer review).
- Geographical inequalities have been reported in biomedical research production, including membership of editorial boards, participation in peer review, and attainment of key authorship positions in submitted and published articles.
- Diversity in science is associated with better research quality and improved representation of global scientists’ opinions.

**WHAT THIS STUDY ADDS**

**HOW THIS STUDY MIGHT AFFECT RESEARCH, PRACTICE OR POLICY**

- Educating faculty mambers, editors, researchers, funders about gender and geographical implicit biases will be key to improve diversity and equity in academia.
- Monitoring sensitive diversity indicators during the editorial decision-making and the peer reviewing processes will help ensure diversity in knowledge and ideas.
- Decentralisation of journal editorial boards, publishing articles in regional languages and creation of independent citation databases at regional level will help to enlarge the diversity of opinions in the scientific literature.
- Providing free or low-cost language support services is another way to improve diversity in research.

## Introduction

 The editorial decision-making process should be impartial and based on the quality of manuscripts, compliance with the journal’s requirements and relevance for its readership; the study results and authors’ characteristics should not be part of the decision to publish. Yet, biases in editorial decision-making have been reported.^1^,^3^ Studies have shown that editorial decision-making is influenced by the methodology used in the reported study (such as the study design, the sample size and the statistical methods used),^4 5^ the strength of the findings,^6^ the source of funding,^4^ positive findings^7^ and whether the corresponding author is affiliated to the same country as the editor of the journal.^4^ Editorial decisions can further be influenced by the perceived high citation potential of the research in a highly competitive editorial market,^8 9^ potential reprint sales revenue^10^ and increased profit from open access business models focusing on volume rather than quality. Some of these elements of a research manuscript are important and easily accessible confounders to be controlled for when assessing the existence of gender and geographical bias during the editorial decision-making process, as they are known to be associated with acceptance for publication.

It is not known whether first and/or last authors’ gender is associated with the chance of getting published. Authors’ gender has been shown to be associated with the reported types of contribution to research projects^11^ and position on the authorship byline of submitted manuscripts.^12^ Moreover, research findings in manuscripts led by male authors were presented more positively compared with those led by female authors,^13^ and this could potentially influence editorial decisions in favour of men first authors. Although some studies concluded the absence of implicit gender biases after finding no difference on acceptance between anonymised versus non-anonymised peer-review processes, they were assessed in a single journal specialised in ecology.^14^

The objectives of the ATHENA case-control study were to assess in a large sample of biomedical journals whether there is an association between the gender of the first (main objective) and last author (secondary objective), and acceptance for publication independently of known factors related to the methods and funding of the research described in the manuscript, for manuscripts sent out for peer review. As many manuscripts are rejected without review, it is also important to assess for gender and geographical bias in these submissions, and this will be explored in a larger cohort study of editorial decisions made on all submitted manuscripts in the same period and journal sample as a further study.

**Figure 1:**
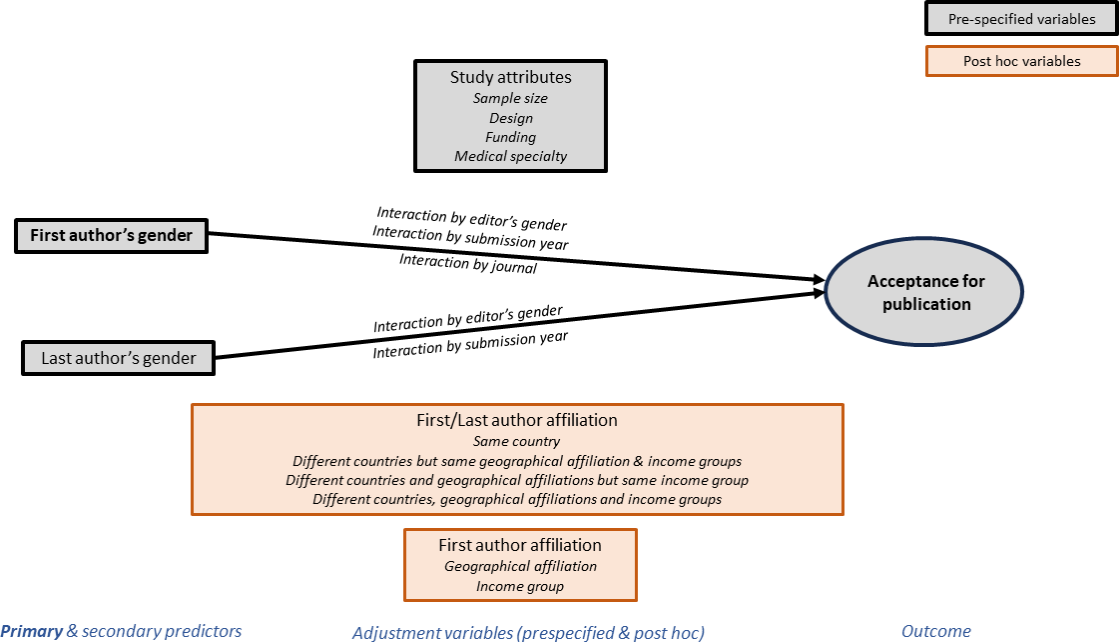
Conceptual framework of the ATHENA project including primary/secondary predictors, pre-specified and post-hoc adjustment variables to model acceptance for publication.

Recently, the geographical location of researchers’ affiliation has been identified as being associated with knowledge dissemination, including authorship positions, publication rates, citation counts and invitation to peer review.^15^,^17^ Certain geographies, such as South America or China, may be subject to bias due to language barriers; they may also be subject to higher levels of prejudice in the perception of lower quality of their research. This might help explain the under-representation of low and middle country-income authors in published articles and subsequently in the citations generated by this research.^15^ The lack of diversity among editors and peer reviewers in terms of gender, geographical affiliation, country-income group, race and ethnicity could also contribute to the paucity of diversity in research publications.^16 17^ Because these studies were published after the writing of our original protocol but prior to our data analyses, we added a new secondary objective to test whether authors’ geographical affiliation and country-income were associated with acceptance for publication in manuscripts sent out for peer review (figure 1), as this outcome had not yet been specifically studied. We also added a post-hoc secondary analysis to see whether peer reviewers’ recommendations of the first version submitted were associated with acceptance for publication.

## Methods

### Study design and settings

**Figure 2:**
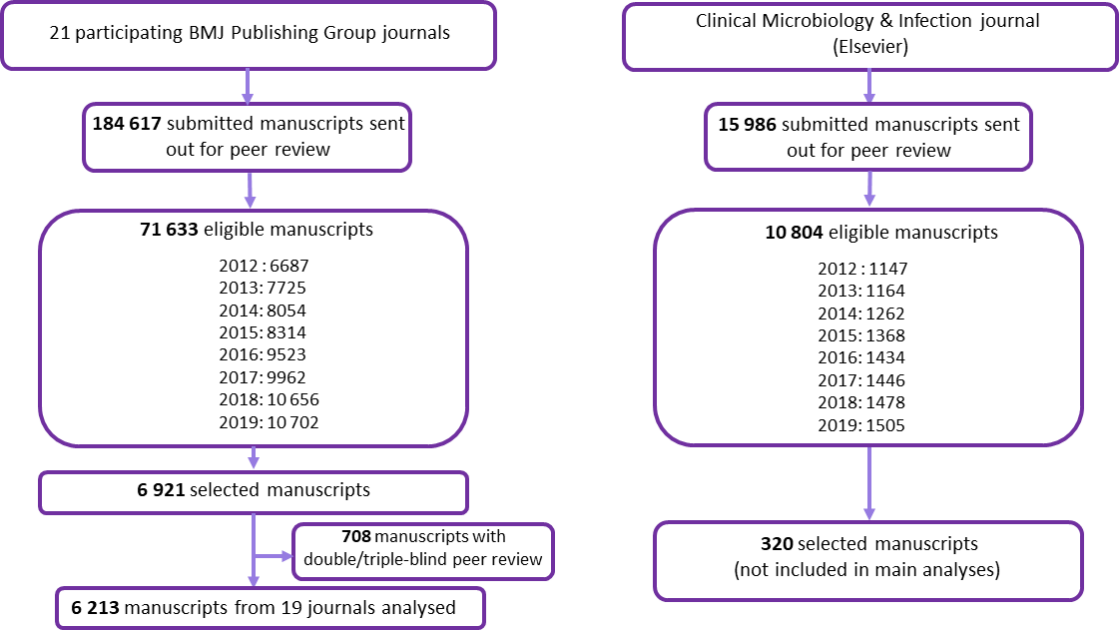
Description of all manuscripts submitted, eligible and selected for ATHENA study between 1 January 2012 and 31 December 2019 by publisher.

The ATHENA case-control study only includes manuscripts sent out for peer review and is nested in a larger prospective cohort study of all original research manuscripts submitted for publication. This case control study initially included 21 biomedical journals from BMJ Publishing Group and one journal from Elsevier—(*Clinical Microbiology & Infection*, CMI), (online supplemental table 1 and figure 2). However, two journals (*Journal of NeuroInterventional Surgery* and *BMJ Quality & Safety*) had to be excluded as they apply specific/unique peer-review processes (double/triple anonymised).

### Participants and data source

We included all original research articles and systematic reviews with multiple co-authors submitted to participating journals between 1 January 2012 and 31 December 2019 (which avoids the effect of the COVID-19 pandemic on research activities), and sent out for peer review, and which had received a final editorial decision by 28 February 2022. We randomly sampled our cases and controls from eligible manuscripts. We extracted data from the manuscript submission systems for BMJ Publishing Group journals, and we received data directly from the CMI database for this specific journal. Data related to the characteristics of the research reported in the first submitted version of these manuscripts were automatically extracted using machine learning from the submitted PDFs by investigators from the Swiss Institute for Bioinformatics (PR, NN, LM, JL, JG). CMI was only included for descriptive purposes and is not included in the main analyses.

### Case and control definition

Cases were manuscripts accepted for publication with at least one completed peer-review report. Controls were manuscripts rejected for publication with at least one completed peer-review report. Accepted and rejected articles (1:1) were randomly selected at the journal level.

### Exposure factors explored and other variables

For each submitted manuscript, we collected details about the journal (impact factor (<5, 5–10, >10) and type of peer-review process (open vs anonymised); manuscript (manuscript ID, title, abstract, original submission date, first decision date, number of co-authors, editorial decision); editor(s)-in-chief (EIC) at the time of submission (first name, last name, gender, country of affiliation); reviewers who provided a recommendation of the first version submitted (first name, last name, country of affiliation); authors (authorship position (where several co-authors held the first or last authorship position we used the first and last declared author respectively), corresponding author status, salutation, first name, middle name, last name, country of affiliation). We classified the countries of affiliation into six geographical affiliation groups (Europe, Africa, Asia, South America, North America or Oceania; Europe & North America for multiple EICs) and further categorised the countries by wealth using the four levels defined by the World Bank Atlas method according to 2021’s gross national country-income per capita (low; lower middle; upper middle and high country-incomes).^18^ For CMI manuscripts, we did not receive data on authors’, reviewers’ and EIC’s country of affiliation, middle names, nor salutation. We categorised the reviewers’ recommendations into four categories (accept, major revisions, minor revisions and reject), except for *The BMJ* journal, for which no recommendation is requested from the reviewers.

#### Journal’s standard process to assess manuscripts submitted for publication

In most journals, once a manuscript has been selected to be sent out for external peer review and a minimum of two review reports have been received, the handling editor (which can be the EIC or an Associate Editor) will receive the recommendations and comments and make a decision on the manuscript. This might include discussing the reviewers’ comments with other editors, members of the journal’s Editorial Board and/or statisticians. The handling editor will then decide based on the reviewers recommendation and the journal editorial line to reject, accept or invite minor/major revisions.^19^

#### Gender determination (main and secondary predictors)

EIC’s gender was provided by BMJ Publishing Group and was estimated for CMI using Gender API based on first name. We used a four-step sequential process to determine authors’ and reviewers’ gender according to ATHENA ancillary studies.^2 16^ First, we used the first name and country of affiliation in Gender API (https://gender-api.com/en) website. Gender API provides gender determination with an accuracy probability from 50% to 100% (under 50% an unknown status is attributed). Second, for undetermined gender, we used middle names and the country of affiliation in Gender API (except for CMI). Third, we used the online service genderize.io (http://genderize.io) to determine gender based on first and middle names which also considers gender as unknown if accuracy is under 50%. Fourth, we used authors’ salutation and attributed with 100% accuracy man to ‘Mr’ or ‘M’ and woman to ‘Miss,’ ‘Mrs,’ and ‘Ms.’. Where determined gender had an accuracy below 80%, we retained the gender determined by salutation and attributed an accuracy of 100%. Finally, we determined gender at three levels of accuracy: ≥60%, ≥70% and ≥80% for all authors and reviewers. We used accuracy ≥80% for all analyses. We cross-tabulated the gender of first and last authors and created the variable ‘gender diversity between first and last author’. For CMI, we were only able to run the first step of the gender determination process for authors and editors. As there were 2–3 EICs for some journals, we coded their gender as ‘Man’, ‘Woman’ or ‘Mixed’.

#### Adjustment variables

We estimated the percentage of female authors among all authors on the byline with a determined gender at three levels of accuracy: ≥60%, ≥70% and ≥80%, and used accuracy ≥80% for all analyses. We created a four-category variable: no female authors, 0%–49%, 50%–99% and 100% female authors.

We created a geographical/income ‘diversity’ variable combining geographical and economic perspectives. We combined the country, geographical affiliation group and country-income group of the first and last authors. This variable is a proxy of diversity in the research team represented by the two main (first and last) authors of the paper.^20^ We created four categories from the ‘lowest level of diversity’ where first and last authors were affiliated to the same country (group 1) to the ‘largest level of diversity’ where both authors were affiliated to different countries, regions and country-income groups (group 4). In-between, we proposed ‘intermediate levels of diversity’: where first and last authors were affiliated to different countries within the same geographical affiliation and country-income groups (group 2); where first and last authors had the same geographical affiliation but different country-income groups (group 3).

#### Study attributes

We used machine learning procedures to identify medical specialty (online supplemental material 2), funding type (online supplemental material 3), study design (online supplemental material 4 and online supplemental table 2) and sample size (online supplemental material 5) of the study reported in the first version submitted of each manuscript.

### Study sample size estimation

We chose an OR of 0.85 to indicate a presence of gender bias towards women for the association between first author’s gender and acceptance for publication. We assumed the proportion of women first authors to be 40% among cases (44% among controls) using OR=0.85. Considering a type-1 error at 5% (two-sided) with a study power of 90% and a correlation coefficient for exposure between matched controls and cases at 0.03 (to consider some clustering at the journal level), we approximated that 2800 cases and 2800 controls were needed. Anticipating 20% missing values or exclusions we increased our sample size to 3500 cases:3500 controls (1:1). We took a random selection of cases and controls by journal, considering 159 journal-years across the study period and 20 cases/20 controls per journal-year corresponding to a maximum of 160 cases/160 controls per journal.

### Statistical analysis

Two journals were excluded from all the analyses. We present continuous variables as means (SD, SD) or medians (25th and 75th percentiles), categorical variables as frequencies and relative frequencies by case/control status. We describe data for CMI journal separately in online supplemental table 3, as we did not have all the data for primary analysis. For a randomly selected set of 100 manuscripts, we evaluated the level of agreement between the studies’ attributes as automatically extracted by machine learning procedures—leveraging the SIB Literature Services (SIBiLS)^21^—compared with a manual extraction performed by consensus between three independent raters (AG-A, KBM, ZM). We calculated unweighted kappa coefficients (ᴋ) for the variables medical specialty and design; weighted kappa coefficient (ᴋw) for the variables funding and sample size. Primary and secondary analyses were conducted for BMJ Publishing Group manuscripts only. For manuscripts with at least two co-authors, we performed unconditional logistic regression models on complete cases with case/control status as the dependent variable. The main predictor was the first author’s gender, and the last author’s gender was a secondary predictor. Initially, the gender of the corresponding author was a secondary predictor, however, in 81% of cases (n=1282), they also held the position of first or last author, so we did not test it. First authors’ gender, prespecified variables (last author’s gender, study attributes) then post-hoc variables (geographical affiliation group and country-income group of first authors, and diversity of authors’ affiliation) were introduced in the main model. The conceptual framework for the selection of variables is shown in figure 1. First authors’ gender was introduced first in the model, followed by the other prespecified variables (last author’s gender, study attributes), then the post-hoc variables (geographical affiliation group, country-income group of first authors and diversity of authors’ affiliation). We prespecified four interaction terms between (1) gender of the EIC and first author; (2) gender of the EIC and last author; (3) submission year and gender of the first author and (4) submission year and gender of the last author. We added a post-hoc interaction between the first author’s gender and the journal to assess the existence of some heterogeneity of the main result due to the inclusion of different journals with various editorial processes. We report unadjusted and adjusted ORs, 95% CI and p values from univariate and multivariable models. We performed three additional sensitivity analyses using levels of accuracy of ≥60%, and ≥70% for gender determination, and a multiple imputation model for missing gender of the first and last authors. Missing gender of the first and last authors was considered at random, and 20 imputations were applied; adjustment of the imputation model was done for geographical affiliation group of the first and last authors, and for the journal. For categorical predictors with more than two modalities, a global p value was computed. We interpreted global p values as significant if p<0.05. As a post-hoc analysis, we calculated the likelihood ratio of the null hypothesis of no gender bias over the alternative hypothesis of presence of gender bias for the association between first author’s gender and acceptance for publication.^22^ We conducted a specific analysis to investigate another stage of the editorial process. We arranged the reviewers’ recommendations into an ordinal scale, ranging from ‘reject’ to ‘major revisions’, ‘minor revisions’ and finally ‘accept’; one journal was excluded from this analysis as it does not ask reviewers to give a recommendation. We considered multiple factors in our analysis, including the gender of both reviewers and the first/last authors, their geographical affiliation, the country-income group and the following attributes of the study: study design, sample size, funding and specialty. To account for the potential clustering of reviewers’ recommendations within manuscripts, we performed multilevel mixed-effects ordered logistic regression models. We used Stata intercooled (STATA, College Station, Texas, USA) V.17.0 for analyses and R software (V.4.1.2) for data management.

### Patient and public involvement

We partnered and co-authored with the chair of BMJ’s LGBTQ+ network (MR). MR contributed to the study conception (definition of primary/secondary objectives), data collection, gender determination method, manuscript revision and final approval. MR will also provide advice for the dissemination of research findings to editors and the public.

## Results

We initially included 7000 manuscripts from BMJ Publishing Group with all pre-specified variables available for analysis but after excluding two journals 6213 manuscripts were analysed (figure 2, table 1). We found a moderate level of agreement between raters and automatic algorithm extraction on medical specialty identification (ᴋ=0.58), funding type (ᴋw=0.54), study design (ᴋ=0.33) and study sample size (ᴋw=0.84).

**Table 1:** Proportion of editorial, authorship and study attributes for 19 BMJ Publishing Group journals by case and control status

Variables	Cases (**n=3119**)	Controls (**n=3094**)	**P** value*
**Authorship**			
First author’s gender† (*missing*), n (%)	*406* (*13.0*)	*513* (*16.6*)	
Man	1399 (51.6)	1384 (53.6)	0.134
Woman	1314 (48.4)	1197 (46.4)	
Last author’s gender† (*missing*), n (%)	*371 (11.9)*	*363 (11.7)*	
Man	1863 (67.8)	1823 (66.8)	0.411
Woman	885 (32.2)	908 (33.2)	
Corresponding author’s gender† (*missing*), n (%)	*412* (*13.2*)	*402* (*13.0*)	
Man	1618 (59.8)	1590 (59.1)	0.597
Woman	1089 (40.2)	1102 (40.9)	
Position of corresponding author (*missing*), n (%)	*45* (*1.4*)	*38* (*1.2*)	
First author	1855 (60.3)	1714 (56.1)	0.003
Last author	654 (21.3)	721 (23.6)	
Other position	565 (18.4)	621 (20.3)	
Gender† diversity first and last authors (*missing*), n (%)	*719* (*23.1*)	*807* (*26.1*)	
Man first author & man last author	897 (37.4)	872 (38.1)	0.581
Woman first author & man last author	724 (30.2)	652 (28.5)	
Man first author & woman last author	349 (14.5)	354 (15.5)	
Woman first author & woman last author	430 (17.9)	409 (17.9)	
Mean number of authors (±SD, Q2: Q1–Q3)	6.7 (±2.8, 7.0: 5.0–9.0)	6.4 (±2.8, 7.0: 4.0–9.0)	<0.001
Mean percentage of female authors (±SD, Q2: Q1–Q3)	41.1 (±25.8, 40.0: 22.2–60.0)	39.6 (±27.2, 40.0: 20.0–57.1)	0.024
Proportion of female authors (*missing*), n (%)	*8* (*0.3*)	*22* (*0.7*)	
0	373 (12.0)	485 (15.8)	<0.001
0–50	1431 (46.0)	1323 (43.1)	
50–100	1179 (37.9)	1116 (36.3)	
100	128 (4.1)	148 (4.8)	
First author’s geographical affiliation (*missing*), n (%)	*38* (*1.2*)	*32* (*1.0*)	
Europe	1623 (52.7)	1381 (45.1)	<0.001
Africa	43 (1.4)	55 (1.8)	
Asia	449 (14.6)	789 (25.8)	
North America	693 (22.5)	584 (19.1)	
Oceania	227 (7.4)	180 (5.9)	
South America	46 (1.5)	73 (2.4)	
Last author’s geographical affiliation (*missing*), n (%)	*8* (*0.3*)	*6* (*0.2*)	
Europe	1625 (52.2)	1391 (45.0)	<0.001
Africa	49 (1.6)	54 (1.7)	
Asia	435 (14.0)	763 (24.7)	
North America	715 (23.0)	617 (20.0)	
Oceania	239 (7.7)	194 (6.3)	
South America	48 (1.5)	69 (2.2)	
First author’s country incomes (*missing*), n (%)	*38* (*1.2*)	*32* (*1.0*)	
High income	2782 (90.3)	2472 (80.7)	<0.001
Upper middle income	211 (6.8)	440 (14.4)	
Lower middle and Low income	88 (2.9)	150 (4.9%	
Last author’s country incomes (*missing*), n (%)	*8* (*0.3*)	*6* (*0.2*)	
High income	2796 (89.9)	2516 (81.5)	<0.001
Upper middle income	224 (7.2)	422 (13.7)	
Lower middle and Low income	91 (2.9%	150 (4.9)	
Geographical/income diversity between first/last authors (*missing*), n (%)	*45* (*1.4*)	*36* (*1.1*)	
Same country	2579 (83.9)	2689 (87.9)	<0.001
Different countries but same geographical affiliation/income groups	175 (5.7)	120 (3.9)	
Different countries and different geographical affiliation but same income group	184 (6.0)	128 (4.2)	
Different countries, geographical affiliation and income groups	136 (4.4)	121 (4.0)	
**Editorial characteristics**			
EIC gender† (*missing*), n (%)	*0* (*0*)	*0* (*0*)	
Man	2249 (72.1)	2204 (71.2)	0.446
Woman	870 (27.9)	890 (28.8)	
EIC’s geographical affiliation (*missing*), n (%)	*0* (*0*)	*0* (*0*)	
Africa	49 (1.6)	60 (1.9)	0.034
Asia	77 (2.5)	42 (1.4)	
Europe	1256 (40.3)	1294 (41.8)	
Northern America	335 (10.7)	327 (10.6)	
Oceania	1049 (33.6)	1023 (33.1)	
Europe+Northern America	353 (11.3)	348 (11.2)	
Editor’s country incomes (*missing*), n (%)	*0* (*0*)	*0* (*0*)	
High income	3070 (98.4)	3034 (98.1)	0.270
Upper middle income	49 (1.6)	60 (1.9)	
Mean number of reviewers providing recommendation‡ (±SD, Q2: Q1–Q3)	2.8 (±1.4, 3.0:2.0–3.0)	2.6 (±1.3, 2.0:2.0–3.0)	<0.001
Reviewers’ gender† (*missing*), n (%)	*830* (*11.0*)	*778* (*11.2*)	0.006
Man	4349 (64.9)	4137 (67.1)	
Woman	2355 (35.1)	2024 (32.9)	
Reviewers’ geographical affiliation‡ (*missing*), n (%)	*861* (*11.4*)	*839* (*12.1*)	0.092
Africa	99 (1.5)	108 (1.8)	
Asia	579 (8.7)	608 (10.0)	
Europe	3422 (51.3)	3040 (49.8)	
North America	2023 (30.3)	1839 (30.1)	
South America	59 (0.9)	63 (1.0)	
Oceania	491 (7.4)	442 (7.2)	
Reviewers’ country income‡ (*missing*), n (%)	*861* (*11.4*)	*839* (*12.1*)	0.704
High income	6241 (93.5)	5706 (93.5)	
Upper middle income	270 (4.0)	251 (4.1)	
Lower middle income	149 (2.2)	126 (2.1)	
Low income	13 (0.2)	17 (0.3)	
Mean interval from submission to first decision (±SD, Q2: Q1–Q3), days	55.8 (±36.2, 48.0: 31.0–70.0)	52.8 (±35.0, 44.0 : 29.0–66.0)	<0.001
**Study attributes**			
Submission year (*missing*), n (%)	*0* (*0*)	*0* (*0*)	<0.001
2012	426 (13.7)	345 (11.2)	
2013	362 (11.6)	407 (13.2)	
2014	367 (11.8)	418 (13.5)	
2015	371 (11.9)	419 (13.5)	
2016	371 (11.9)	433 (14.0)	
2017	392 (12.6)	373 (12.1)	
2018	435 (13.9)	347 (11.2)	
2019	395 (12.7)	352 (11.4)	
Study design (*missing*), n (%)	*0* (*0*)	*0* (*0*)	0.077
Randomised controlled trial	192 (6.2)	152 (4.9)	
Basic sciences studies	136 (4.4)	96 (3.1)	
Case-control study	77 (2.5)	78 (2.5)	
Cohort study	1469 (47.1)	1496 (48.4)	
Comparative study	267 (8.6)	289 (9.3)	
Cross-sectional study	447 (14.3)	458 (14.8)	
Medico-economics/simulation study	35 (1.1)	26 (0.8)	
Mixed/qualitative methods	389 (12.5)	385 (12.4)	
Systematic review and meta-analysis	107 (3.4)	114 (3.7)	
Sample size (*missing*), n (%)	*0* (*0*)	*0* (*0*)	0.206
≤100 or no sample size	1162 (37.3)	1197 (38.7)	
101–1000	1006 (32.3)	1017 (32.9)	
1001–10 000	555 (17.8)	537 (17.4)	
>10 000	396 (12.7)	343 (11.1)	
Type of funding§ (*missing*), n (%)	*0* (*0*)	*0* (*0*)	<0.001
Non-profit only	596 (19.1)	611 (19.7)	
Associations and foundations	213 (6.8)	178 (5.8)	
For profit	97 (3.1)	92 (3.0)	
International organisations	146 (4.7)	75 (2.4)	
Non-profit & associations and foundations	775 (24.8)	657 (21.2)	
Non-profit & for-profit	123 (3.9)	107 (3.5)	
No information of the funding	891 (28.6)	1023 (33.1)	
No funding	278 (8.9)	351 (11.3)	

* P values from the univariate analyses (logistic regression models, except for the continuous variables where comparisons were done using Student’s t-test).

† Gender identified with an accuracy above 80%.

‡ For cases, n=7534 reviewers, for controls, n=6939 reviewers.

§ Funding type is detailed in online supplemental material 3.

### Proportion of editorial, authorship and manuscript characteristics by accepted and rejected status

The distribution of EIC’s geographical affiliation was significantly different between accepted and rejected manuscripts (higher proportion of EICs affiliated to Asia among accepted than rejected), and the mean duration from submission to first decision was on average 3 days longer for accepted than rejected (table 1). Accepted manuscripts received more reviewers’ recommendations on the first version submitted than rejected manuscripts; there were more women reviewers among accepted than rejected manuscripts, but reviewers’ geographical affiliation and country-income group did not significantly differ between accepted and rejected manuscripts (table 1). However, overall, the reviewers who completed the review were more often men (66.0%), and more often affiliated to a high country-income group (93.5%). The proportion of women first authors was 2.0 points higher, and the overall percentage of women co-authors 1.5% higher in accepted than in rejected. Only the difference in mean percentage of female authors was statistically significant between accepted and rejected. Accepted manuscripts had on average +0.3 co-authors compared with rejected. There were significantly more first and last authors with European, North American or Oceanian affiliations among accepted than rejected; first and last authors were more often affiliated to a high country-income among accepted than rejected. The proportion of manuscripts with different geographical affiliation groups or country-income groups between first and last authors was significantly higher for accepted compared with rejected. Regarding the manuscript attributes, the proportion of studies stating no funding or where funding was not declared was significantly lower among accepted than rejected. The proportion of accepted manuscripts was significantly higher in 2012 compared with later years.

### Association between gender of the authors and acceptance for publication (primary objective)

Table 2 shows univariate and multivariable analyses of the main analysis. The association between gender of the first author and acceptance for publication remained non-significant once funding, and medical specialty was introduced in the model; it stayed non-significant after adjustment for the post-hoc variables (geographical affiliation group and country-income group of first authors and diversity of authors’ affiliations). Gender of the last author was not associated with acceptance for publication in the univariate and multivariable analyses. The interactions between the gender of the EIC and first author (adjusted OR woman 1.21, 0.92 to 1.58, p=0.170), and between the gender of the EIC and last author (woman 1.17, 0.88 to 1.55, p=0.278) were both non-significant. The interactions between the year of submission and respectively the gender of first (p=0.872) and last authors (p=0.436) were non-significant. Finally, the interaction between the first author’s gender and the journal was non-significant (p=0.116). We found similar findings with the sensitivity analyses (online supplemental table 4). Acceptance for publication was also independently associated with type of funding (higher for studies funded by international organisations adjusted OR 2.76, 95% CI 1.87 to 4.10, non-profit & associations & foundations (1.30, 1.04 to 1.62) and associations & foundations (1.40, 1.04 to 1.89) compared with studies declaring no funding) (table 2). The likelihood ratio of no gender bias (null hypothesis) over existence of gender bias (alternative hypothesis) was 182, providing decisively strong evidence in favour of the null hypothesis.

**Table 2:** Acceptance for publication in a random sample of 6213 accepted/rejected manuscripts submitted to 19 BMJ Publishing Group journals between 1 January 2012 and 31 December 2019 with at least two coauthors on the byline. Univariate and multivariable models*†

Independent variables	Univariate analyses			Multivariable analysis*^†^		
	OR	**95% CI**	**P** value	OR	**95% CI**	**P** value
**Author/editor characteristics**						
First author’s gender‡ (ref=Man)			0.134			0.578
Woman	1.09	(0.98 to 1.21)		1.04	(0.92 to 1.17)	
Last author’s gender† (ref=Man)			0.411			0.451
Woman	0.95	(0.85 to 1.07)		0.95	(0.84 to 1.08)	
Gender diversity between first and last authors (ref=men first & last)			0.5811	–	–	–
Woman first author & man last author	1.08	(0.94 to 1.24)	0.288			
Man first author & woman last author	0.96	(0.81 to 1.14)	0.634			
Woman first author & woman last author	1.02	(0.87 to 1.21)	0.795			
Proportion of women co-authors on byline‡ (ref=only men)			<0.001	–	–	–
Up to 49% women	1.41	(1.21 to 1.64)	<0.001			
From 50 to 99% women	1.37	(1.17 to 1.61)	<0.001			
100% women	1.12	(0.86 to 1.48)	0.398			
First author’s geographical affiliation^e^ (ref=Europe)			<0.001			<0.001
Africa	0.67	(0.44 to 0.99)	0.049	0.75	(0.41 to 1.36)	0.345
Asia	0.48	(0.42 to 0.56)	<0.001	0.58	(0.46 to 0.73)	<0.001
North America	1.01	(0.89 to 1.15)	0.885	1.01	(0.86 to 1.17)	0.947
Oceania	1.07	(0.87 to 1.32)	0.507	1.21	(0.95 to 1.54)	0.116
South America	0.54	(0.37 to 0.78)	0.001	0.68	(0.40 to 1.16)	0.157
First author’s country of affiliation income (ref=high income)			0.001			0.047
Upper middle income	0.43	(0.3 to 0.51)	<0.001	0.66	(0.47 to 0.95)	0.024
Lower middle and low income	0.52	(0.40 to 0.68)	<0.001	0.69	(0.46 to 1.03)	0.072
Last author’s geographical affiliation (ref=Europe)			<0.001	–	–	–
Africa	0.78	(0.52 to 1.15)	0.208			
Asia	0.49	(0.43 to 0.56)	<0.001			
North America	0.99	(0.87 to 1.13)	0.903			
Oceania	1.05	(0.86 to 1.29)	0.607			
South America	0.60	(0.41 to 0.87)	0.007			
Last author’s country of affiliation income (ref=high income)			<0.001	–	–	–
Upper middle income	0.48	(0.40 to 0.57)	<0.001			
Lower middle and low income	0.55	(0.42 to 0.71)	<0.001			
Geographical/income diversity between first/last authors (ref=same country)			<0.001			<0.001
Different countries, but same geographical affiliation/income group	1.52	(1.20 to 1.93)	0.001	1.35	(1.03 to 1.77)	0.031
Different countries and geographical affiliation but same income group	1.50	(1.19 to 1.89)	0.001	1.50	(1.14 to 1.96)	0.004
Different countries, geographical affiliation and income groups	1.17	(0.91 to 1.51)	0.215	1.78	(1.27 to 2.50)	0.001
EIC’s gender (ref=Man)			0.446	–	–	–
Woman	0.96	(0.86 to 1.07)				
EIC’s geographical affiliation (ref=Europe)			0.034	–	–	–
Africa	0.84	(0.57 to 1.24)	0.38			
Asia	1.89	(1.29 to 2.77)	0.001			
North America	1.06	(0.89 to 1.25)	0.536			
Oceania	1.06	(0.94 to 1.19)	0.353			
Europe & North America	1.05	(0.88 to 1.24)	0.605			
EIC’s country of affiliation income (ref=high income)			0.270	–	–	–
Upper middle income	0.81	(0.55 to 1.18)				
**Study attributes**						
Submission year (ref=2012)			<0.001	–	–	–
2013	0.72	(0.59 to 0.88)	0.001			
2014	0.71	(0.58 to 0.87)	0.001			
2015	0.72	(0.59 to 0.88)	0.001			
2016	0.69	(0.57 to 0.88)	<0.001			
2017	0.85	(0.70 to 1.04)	0.115			
2018	1.02	(0.83 to 1.24)	0.882			
2019	0.91	(0.74 to 1.11)	0.353			
Study design (ref=Randomised controlled trial)			0.077			0.056
Basic sciences studies	1.12	(0.80 to 1.57)	0.505	1.48	(0.96 to 2.27)	0.076
Case-control study	0.78	(0.53 to 1.14)	0.204	0.88	(0.57 to 1.36)	0.556
Cohort study	0.78	(0.62 to 0.97)	0.028	0.78	(0.60 to 1.02)	0.069
Comparative study	0.73	(0.56 to 0.96)	0.023	0.79	(0.57 to 1.08)	0.143
Cross-sectional study	0.77	(0.60 to 0.99)	0.043	0.78	(0.58 to 1.05)	0.095
Medico-economics/simulation study	1.07	(0.62 to 1.85)	0.821	0.91	(0.49 to 1.72)	0.78
Mixed/qualitative methods	0.80	(0.62 to 1.03)	0.086	0.83	(0.61 to 1.12)	0.216
Systematic review and meta-analysis	0.74	(0.53 to 1.04)	0.086	0.79	(0.53 to 1.17)	0.236
Sample size (ref ≤100 or no sample size)			0.206			0.063
101–1000	1.02	(0.90 to 1.15)	0.756	1.14	(0.98 to 1.31)	0.081
1001–10 000	1.06	(0.92 to 1.23)	0.392	1.07	(0.90 to 1.29)	0.43
>10 000	1.19	(1.01 to 1.40)	0.04	1.31	(1.06 to 1.62)	0.012
Type of funding (ref. No funding)			<0.001			<0.001
Associations and foundations	1.51	(1.17 to 1.95)	0.001	1.40	(1.04 to 1.89)	0.025
For-profit	1.33	(0.96 to 1.84)	0.085	1.24	(0.85 to 1.82)	0.257
International organisations	2.46	(1.79 to 3.38)	<0.001	2.76	(1.87 to 4.10)	<0.001
No information of funding	1.10	(0.92 to 1.32)	0.304	1.08	(0.88 to 1.34)	0.459
Non-profit	1.23	(1.02 to 1.50)	0.035	1.20	(0.95 to 1.51)	0.13
Non-profit & associations & foundations	1.49	(1.23 to 1.80)	<0.001	1.30	(1.04 to 1.62)	0.023
Non-profit & for-profit	1.45	(1.07 to 1.97)	0.016	1.21	(0.84 to 1.76)	0.303

* Model performed for manuscripts with more than one author (n=4677 observations).

† Model adjusted for the specialty of the research topic (p=0.675).

‡ Gender determined with accuracy ≥80%.

### Association between geographical affiliation of the authors and acceptance for publication (post-hoc objective)

Acceptance for publication was significantly associated with geographical affiliation group of the first author: compared with Europe, the adjusted OR was significantly lower for first authors affiliated to Asia (0.58, 0.46 to 0.73) (table 2). The likelihood of acceptance was significantly lower when first author was affiliated to upper-middle (0.66, 0.47 to 0.95) compared with high country-income group. We found similar results when using the affiliation group of the last author, except that the odds for publication acceptance were not statistically different for the comparison between Africa and Europe (data not shown). Compared with when first and last authors were affiliated to the same countries, the OR was higher than 1 when both were affiliated to different countries but the same geographical and country-income groups (adjusted OR 1.35, 1.03 to 1.77), when both were affiliated to different countries and geographical affiliations but the same country-income group (1.50, 1.14 to 1.96) or when both had different countries, geographical affiliation and country-income groups (1.78, 1.27 to 2.50) (table 2).

### Association between reviewers’ recommendations and the first/last authors’ gender and study attributes (ad hoc analysis)

In the univariate analyses, reviewers’ recommendation for publication was not significantly associated with the first/last authors’ gender. However, it was strongly associated with final acceptance for publication (crude OR 5.36, 4.98 to 5.78), and associated with first/last authors’ geographical affiliation and country-income group, and geographical/income diversity of first/last authors’ affiliations (online supplemental tables 5 and 6). The proportion of men reviewers was greater for recommendations to accept (67.1%) or reject (68.2%) the manuscript than to provide major (66.1%) or minor (64.3%) revisions; the proportion of women reviewers was greater for recommendations to provide major (33.9%) or minor revisions (35.7%) than to accept (32.9%) or reject (31.8%). Reviewers affiliated to Africa recommended acceptance more frequently than rejection (3.6% vs 1.0%) while reviewers affiliated to Europe recommended rejection more frequently than acceptance (50.7% vs 47.8%). Reviewers with affiliations in the high country-income group recommended rejection more often than acceptance (94.7% vs 88.6%), while it was the opposite for reviewers affiliated to countries in the upper middle (3.5% vs 6.3%), and lower middle country-income groups (1.6% vs 4.7%). Type of funding and medical specialty were also significantly associated (p<0.001, online supplemental table 5) with reviewers’ recommendation. In the multivariate analyses (online supplemental table 7), the variables which remained significantly associated with reviewers’ recommendations were first authors’ geographical affiliation (lower adjusted OR for Asia 0.65, 0.56 to 0.76 compared with Europe); geographical/income diversity in the research team (higher adjusted OR for different geographical affiliation but same country-income group 1.33, 1.08 to 1.65 compared with same country); reviewers’ country-income group (1.80, 1.25 to 2.59 for lower middle- compared with high country-income group); the type of study funding (1.71, 1.27 to 2.29 for funding by international organisation compared with no funding) and medical specialty (see online supplemental table 7).

## Discussion

Manuscripts with a woman first author sent out for peer review were not associated with lower manuscript acceptance as hypothesised. Nor was the gender of the last author associated with acceptance for publication. Post-hoc calculation of the likelihood ratio provided decisive evidence supporting the absence of gender bias in publication acceptance. In the additional analyses, the probability of being accepted for publication, for papers sent out for peer review, was lower when the first author was affiliated to an Asian country compared with a European country, and when first author was affiliated to an upper-middle or lower-middle/low-income compared with a high-income country group. Nevertheless, more geographical/income diversity between first/last authors was associated with a higher probability of acceptance. Type of funding was also independently associated with acceptance for publication. We found the same findings regarding the association between the first and last authors’ gender, the first author’s geographical affiliations and country-income groups and geographically diverse research team and the reviewers’ recommendations for publication.

### Comparison with other studies

The fairness of publication decisions relies on an ecosystem that is not gender-balanced with a higher proportion of men among editors and the assignment of more submissions to men peer reviewers.^23^ Similar to ours, previous studies^424^,^27^ also did not find an association between the gender of key authorship positions and acceptance for publication. Those studies were mainly based on a single medical specialty and used different research methods none of which adjusted for the authors’ and study’s attributes.^28^ Similar to our findings, Burns *et al* demonstrated that papers with a first author affiliated to Asia, Africa or South America had a lower chance of acceptance for publication than those with authors affiliated to North America.^26^ Similarly, Rivadeneira *et al* showed that manuscripts with authors from North America and Oceania had greater odds of acceptance than those from Europe or Asia.^27^ The impact of the diversity of authors’ affiliations on manuscript outcomes has been little studied in science. However, Campbell *et al* explored submissions and publications across American Geophysical Union journals from 2012 to 2018 in terms of national affiliation, gender, career stage of individual authors and race/ethnicity for US-based authors.^29^ Acceptance was +2.8% higher for cross-cultural collaborations compared with international teams, and +4.5% higher for mixed-gender compared with single-gender teams. In line with our findings, they found no significant difference in acceptance rates between manuscripts with a woman or man first author. Although they used a more intersectional method for defining diversity, they too found diverse teams to have higher acceptance rates than non-diverse teams.

### Study strengths and limitations

The inclusion of submissions from 19 BMJ Publishing Group journals over 8 years, including two large general medical journals, and journals in a range of biomedical specialties attracting submissions from around the world provided a rich and diversified sample. While inclusion of journals from BMJ Publishing Group alone, which has a commitment to improving equality and diversity,^30^ limits the generalisability of the findings to other journals, we sampled manuscripts submitted from 2012 to 2019. Back then, equality, diversity and inclusion were less of a focus for publishers than in recent years,^30 31^ particularly since the Royal Society of Chemistry launched the Joint Commitment for Action on Inclusion and Diversity in Publishing in 2020 in response to the Black Lives Matter movement.^32^ Although our study initially planned to include more biomedical journals from other publishers, we only managed to include one other journal which could not provide the same set of data as the BMJ Publishing Group so we presented this data separately.

To limit information bias, we chose a case-control design and randomly selected cases and controls from eligible manuscripts using a computerised procedure. To avoid potential confounders from the case-control study, we evaluated the presence of gender and geographical bias only in manuscripts sent out for peer review. However, a substantial proportion of submissions that were not sent to peer review remain unexplored and may have been more prone to gender bias. This will be fully investigated in a further retrospective cohort study of all research submissions to participating journals during the same period.

In the absence of self-identified data from authors, we determined gender as binary and by doing so may have misrepresented people with diverse gender identities. However, we applied a four-step sequential procedure already implemented in our two previous works^12 16^ and used the threshold accuracy of gender determination above 80% for our primary analysis. Our three sensitivity analyses using lower thresholds of accuracy for gender determination and multiple imputation to replace missing gender data were in line with our original results.

Unlike previous studies on gender and geographical bias,^15^,^1728^ we adjusted for author and study attributes which are important confounders. However, we acknowledge that the quality of a study and its manuscript is a major factor influencing publication acceptance and could be associated with many of our predictors, making it an important confounding variable. However, in the absence of a rigorous tool applicable to all study designs, we could not robustly assess manuscript quality for such a large set of manuscripts. We used an algorithm trained with machine learning to automatically extract study attributes as it was not feasible to do this manually for such a large sample of manuscripts but the agreement between raters and the automatic algorithm extraction of variables was only moderate. Discrepancies between manual and automatic extractions were randomly distributed and should not have biased the estimated association between study attributes and the outcome. However, the findings that manuscripts on basic sciences were more often accepted for publication than randomised trials should be interpreted cautiously, because the level of agreement between raters and automatic algorithm extraction was low for study design and we might suspect an information bias.

Defining diversity is complex as it incorporates various intersectional dimensions. We explored a definition of research team diversity by combining information from two covariates (geographical affiliation and country-income group of the first and last authors’ country of affiliation) and by adjusting for their gender. This definition is limited as it is not based on the affiliation of all co-authors and only included the first institutional affiliation for an author if there were multiple so teams may have been more diverse than what we captured, so our variable is more a proxy of the diversity of the research team. Moreover, we used the 2021 World Bank classification to examine geographic bias and did not account for variations in country classifications across years, we may have diluted the association between country income of first/last authors and editors and acceptance for publication.

### Policy implications

The fact that we did not identify evidence of gender inequalities during the editorial decision-making process and reviewers’ recommendations for manuscripts sent out for review may be ‘reassuring’, but gender bias in research production still deserves further attention. There has been a discrepancy for several decades between near sex/gender equity in medical school and graduate institutions in Europe and North America and the existence of gender inequalities in the research production (46.0% and 31.4% women among respectively first and last authors in 2018–2019, respectively 29.4% and 21.1% in COVID-19-related manuscripts during early pandemic).^12^ Our results need to be confirmed in a randomised trial where the influence of known and unknown confounding factors can be accounted for. The limited geographical representation among first authors in accepted manuscripts heightens the potential for an inadequate reflection of the opinions of global scientists. More crucially, it may also result in a lack of representation of patients and populations requiring prevention and care.^15 16^ The determinants and implications of lower acceptance of manuscripts from Asian geographical affiliations, and middle to low country-income groups and the intersection with authors’ gender need to be further explored to better understand the root cause of the well-established gender inequalities in authorship. Research needs gender and geographical diverse teams^33^ and equitable partnerships with low-resource countries,^34^ because they lead to greater visibility (in terms of citations) and high-quality research.^21^ Our finding of a positive effect of geographically and gender diverse research teams on publication acceptance has been demonstrated by others^35^,^38^ but this could also reflect ‘domestic helicopter research’ practices where researchers from high-income countries take the more senior authorship positions.^39^ Subjective and hidden factors, such as implicit cognitive biases related to geographical affiliations, might also drive perceptions of study quality and the judgements of peer reviewers and subsequently the final editorial decision, however, they are more difficult to investigate.^40^ Anonymising peer review has been proposed to reduce bias against authors but a meta-analysis has shown that it does not affect the quality of peer reviewers’ reports^41^; and AI also has the potential to undo the anonymity of blinded peer review.^42^ Moreover, open and published peer review which aim to restore trust in the integrity and fairness of the review process through transparency to readers have also been shown not to affect the quality of review.^43^ In addition, double anonymising the peer-review process has not been shown to improve geographic diversity among authors,^44^ but triple-anonymised peer review (where even the handling editor of a manuscript cannot see the identity of the authors) has provided some evidence of reducing editorial bias in a cardiology journal.^45^ Educating faculty members about implicit biases and strategies for overcoming them has provided positive effects on implicit biases surrounding women and leadership in academic medicine^46^ and should be extended to geographical implicit bias. As gatekeepers of publication decisions, editors play a crucial role in improving global diversity in research through the acknowledgement of their own potential implicit biases by monitoring sensitive diversity indicators for their peer reviewers and the authors of manuscripts they choose to reject or peer review, to help ensure diversity of knowledge and ideas.^47^ They can also promote the systemic detection of implicit biases in reviewers’ comments, editors’ decisions and the content of the authors’ research itself as recently advocated by *Nature*.^48^ Linguistic bias is another barrier for non-native English-speaking researchers, especially from low or middle country-incomes, and can lower the chance of acceptance for publication.^43 49^ Other possible solutions to facilitate the publication of high-quality research in regional languages include the decentralisation of editorial boards, by including, for example, South/Latin American or Chinese board members, publishing articles in regional languages or decentralising the indexing of journals by creating independent citation databases at regional level (for eg, the African Citation Index).^50^ Provision of free or low-cost language support services promoted by high country-income universities or other public academic institutions and designed for non-native English-speaking authors is thus desirable.^51^ Some journals are starting to experiment with AI-powered scientific writing assistants,^52^ and uptake and beneficial effects of using these should be monitored.

## Conclusions

The lack of evidence gender bias in the final editorial decisions of peer-reviewed papers in the sampled journals may be reassuring. However, the over-representation of high-income countries among authors of papers accepted for publication, reviewers and EICs all support demands for improving equity, diversity and inclusion in biomedical research. This finding underscores the importance of maintaining rigorous and fair review processes, ensuring that research is evaluated solely on its merit. Research needs diverse teams and equitable partnerships with low-resource countries, yet inclusivity in biomedical research is currently insufficient. Funders, academic institutions, researchers, biomedical journal editors and publishers are key to achieving this goal.

## Supplementary material

10.1136/bmjebm-2024-113083 online supplemental file 1

10.1136/bmjebm-2024-113083 online supplemental file 2

## Data Availability

Data are available in a public, open access repository.
