# Supplementary material for: Gender and geographical bias in the editorial decision-making process of biomedical journals: a case-control study
Source: BMJ Evid Based Med. 2024 Dec 25;30(3):e113083. doi: 10.1136/bmjebm-2024-113083 (PMC12171469; doi:10.1136/bmjebm-2024-113083)
Supplement: online supplemental file 2 [file bmjebm-30-3-s002.pdf]

The ATHENA study: Assessment of gender bias in editorial decision-making: Does first auTHor gENder have an effect on manuscript Acceptance? An observational study of medical journals.

Short title: First author's gender and acceptance for publication

**Principal investigator**	Prof. med. **Angèle Gayet-Ageron**, MD, MPH, D<br>Division of Clinical Epidemiology, Medical Directorate<br>University hospitals of Geneva<br>Rue Gabrielle Perret-Gentil 4<br>1211 Geneva 14, Switzerland<br>Phone +41 79 553 4650<br>Email : angele.gayet-ageron@hcuge.ch
**Co-investigators**	Dr. med. **Angela Huttner**, MD, PD<br>Division of Infectious Diseases<br>Department of Internal Medicine<br>Rue Gabrielle-Perret-Gentil 4<br>Geneva, Switzerland 1205<br>Phone +41 79 553 3396<br>Email: angela.huttner@hcuge.ch<br><br>**Dr. Sara Schroter**<br>Senior researcher<br>BMJ Publishing Group, London, UK<br><br>**Mark Richards**<br>Article Transfer Service Manager<br>BMJ Publishing Group, London, UK
**Postdoctorate**	Dr. **Khaoula Ben Messaoud**, PhD<br>Division of Clinical Epidemiology, Medical Directorate<br>University hospitals of Geneva<br>Rue Gabrielle Perret-Gentil 4<br>1211 Geneva 14, Switzerland

	Email: khaoula.benmessaoud@hcuge.ch
**Partners**	**Dr Peter Donnelly**, BSc PhD FRCPath<br>Editor in Chief<br>Journal of Antimicrobial Chemotherapy<br><br>**Colin Drummond**<br>Journal of Antimicrobial Chemotherapy<br><br>**Julia Friedman**<br>Editorial Manager<br>Clinical Microbiology and Infection<br><br>**Prof. Leonard Leibovici**<br>Editor-in-Chief<br>Clinical Microbiology and Infection
**Sponsor**	University of Geneva, Faculty of Medicine
**Sponsor representative**	University of Geneva, Faculty of Medicine<br>Department of health and community medicine, University of Geneva<br>Prof. med. **Samia Hurst**, MD, PhD
**Funding source**	Swiss National Science Foundation (no. 10001A_192374 / 1)
**Protocol version**	V4 (modified on March 23, 2022)
**Modified**	V4_3 (February 15, 2024)

1. Summary of the research plan

1.1. Background and rationale

In recent decades, the proportion of women medical students has increased worldwide and has now outmatched their representation in the general population. This contrasts with the underrepresentation of women in leadership positions. In Switzerland, only 12% of senior doctors heading a medical service in 2018 were women; only 18% of full or associate professors in Swiss Universities were women. There are various reasons for this gender imbalance, including difficulties managing academic career, relationships and family. Another reason has been gender discrimination against women as their careers progress, such as in the selection of future medical specialists, the offer of mentoring, or the awarding of grants. Gender imbalance has also been demonstrated in contributions to research; men are more frequently assigned tasks considered more prestigious, whereas women more frequently receive operational tasks. These differences explain why women are less often at the strategic last-author position and more frequently in a less valued position. While we have identified an apparent gender gap during the research process, to our knowledge, no one has yet explored the existence of gender bias at a later point in the pipeline: specifically, during the editorial decision-making process, once the results of the research have been submitted for publication. A potential gender bias at this level could also explain why women have lower h-indices and, as a result, are still underrepresented among professors in universities. Geographical bias can also be suspected during the editorial decision-making process as it was highlighted in one ancillary study to the Athena project, and it will be also investigated as a post-hoc assessment.

1.2. Overall objectives and specific aims

Our primary objective is to study the association of first authors' gender with acceptance for publication of original research articles sent for peer review. Our secondary aims are also 1) to study the association of last authors' gender with acceptance for publication of original research articles sent for peer review; 2) to examine the existence of a geographical bias during the editorial decision-making process; 4) to assess the existence of an independent gender bias against first authors at key decision points (first and last decision by editors, peer reviewers' score and recommendations) in the editorial decision-making process from initial submission to final decision.

1.3. Methods

This will be a case-control study nested in a retrospective cohort of original articles submitted to medical journals during an 8-year period. The primary outcome will be women's (or men's) rate of acceptance for publication after peer review. The main exposure variable will be the first author's gender. Corresponding and last authors' gender will also be assessed in secondary analyses. Other exposure factors will be year of publication and key variables related to the research described in the manuscripts, as well as editors' and reviewers' gender. We will also assess the geographical affiliation of the first and last authors, and of the editor as other exposure factors associated with the acceptance for publication. Finally, we will assess if diversity in the research team, defined by any differences in the countries and geographical affiliations of the first and the last authors is associated with the acceptance for publication. The handling editor's gender will be assessed as an effect modifier in the association between the first author's gender and acceptance for publication.

1.4. Expected results and their impact

If we observe that the likelihood of acceptance is at least 0.85 (odds ratio) for women compared to men first authors, it will contribute to our understanding of why women have lower publication rates and thus make slower

progress in academia. Thereafter interventions, such as editor anonymising regarding the author's name, could be assessed. Other methods are already implemented in some journals, such as fully open peer review journals where authors and reviewers are named, and this might impact on results as well. If we do not find gender bias in editors' and reviewers' decision-making, we will be able to conclude that the imbalance in gender inequality is more likely due to discrimination upstream to the editorial decision-making step. This would justify the continuation and expansion of institutional initiatives for mentorship and promotion of women's careers in sciences and unconscious bias training for editors as well as the importance of journals collecting and reporting on the gender of authors and peer reviewers.

2. Research plan

2.1. Current state of research in the field

In recent decades, the proportion of women medical students has increased worldwide and has even outmatched their representation in the general population.[1] This contrasts with the underrepresentation of women in leadership and academic positions. According to the Swiss Medical Association (FMH), only 12% of senior doctors heading a medical service in 2018 were women.[2] In 2012, only 18% of full or associate professors in Swiss Universities were women.[3] Local demographic statistics at the Faculty of Medicine of the University of Geneva (UNIGE) are in line with worldwide findings on the underrepresentation of women among professors. While for the last two decades more than 60% of students have been women, the proportion of women professors at UNIGE is still dramatically low, at 6% in 2000, 16% in 2010, and 21% in 2015.[4] Various reasons might explain this gender imbalance, the first being that women face greater difficulties in managing an academic career, partnership and family. Women more frequently put family duties before their careers.[3] Another reason (and not the least) is the existence of gender bias at different stages of medical studies and academic training. During medical school, gender and generation are both important influences on career and specialty choices.[5–7] Thereafter, women face more gender discrimination and have more difficulties than men in finding a mentor to help manage their career and facilitate their advancement and productivity.[8] Women scientists are also less likely to get funded for their research due to a less favourable assessment of grants written by women principal investigators and not due to differences in the quality of proposals led by men and women.[9] In another experiment, gender bias against women was demonstrated in the assessment of student application materials for a laboratory manager position when they were randomly assigned either a male or a female name.[10] Finally, in the area of medical publication, a gender imbalance is striking: only 33% and 18% of first and last authors, respectively, are women.[11,12] Though the representation of women first authors increased significantly between 2000 and 2015, it still remains well below 50% and thus does not reflect the current "feminisation" of the medical profession.[13] During the early period of pandemic, we observed increased gender disparities in attainment of co-authorship positions on submitted research related to covid-19 which narrowed in the second year of the pandemic.[14] Because academic promotion and awarding of research grants are heavily influenced by the publication records of candidates, and competition for prominent authorship positions (first, last, corresponding) is high, there is an urgent need to take into account the inequalities in terms of scientific production to the detriment of women. This helps explain the gender gap against women regarding their scientific productivity and thus their difficulties to be promoted.[12] Beyond the medical sciences, the influence of gender on peer review outcomes has also been assessed and has revealed contrasting

results.[15–17] We recently reported a double gender and geographical bias in the invitation and participation to the peer review process.[18] Women are less invited to peer review (39% women among invited peer reviewers) compounded by fewer women agreeing to peer review invitations (31% women agreed vs. 38% of men). Reviewers with African, Asian and South American affiliations and those from low- and middle-income countries are underrepresented, even whey they were more likely to agree to review.

With regards to the division of labour in research, similar to the findings from other studies,[19] our analysis of published article contribution statements found there are significant differences between men and women authors: men are more frequently assigned tasks considered more prestigious, such as conception and study design, fundraising, data analysis, and paper writing, while women are assigned more operational tasks such as data collection, logistics and administrative support.[13] These differences could explain why women are less likely to be last author compared to men as reported elsewhere.[12] Women were more frequently second authors and less likely next-to-last authors compared to men.[13] Some characteristics of submitted manuscripts, such as study design, analytical methods, disclosure of funding source and country where the research took place were also independently associated with acceptance for publication.[20] It is also possible that within research collaborations, women are frequently offered first or last authorship on secondary less important and lower-quality papers.[12] If this were the case, we would expect to find that women are more frequently authoring papers of lower quality or that women are more frequently first authors of papers presenting results from ancillary or *post-hoc* studies. This would also explain why women have fewer publications and citation rates compared to men, and thus have lower h-indices.

While we have identified a gender gap generally in the research process, to our knowledge, no studies have yet explored specifically the existence of gender bias at the final stage of the publishing process: when the editor of a medical journal decides to accept or decline a submitted research manuscript. The existence of geographical and income-country bias has not been further investigated in the acceptance for publication.

2.2. Current state of your own research

In 2015, we assessed professors' h-indices in a local survey of UNIGE's Faculty of Medicine and showed that they were significantly lower among women (mean 22.4±15.1 versus 32.0±10.3, p=0.001), whether in clinical medicine or basic sciences. This discrepancy persisted throughout a period during which several initiatives for promoting women in academia had been launched. Among clinical-medicine professors, women published significantly fewer papers than their male counterparts at the time of the assessment (mean 92 papers ±59 versus 163 ±107, p<0.001). The results of this survey were not published; the manuscript was rejected from seven peer-reviewed journals. The main criticism was a (perceived) lack of generalizability of its findings (personal communication). The study dataset is, however, available in a public repository (https://easy.dans.knaw.nl/ui/datasets/id/easy-dataset:76777). Though never published, its results triggered the implementation of studies aiming to better understand the drivers of gender inequity in scientific productivity and thus to promote women more effectively in academia. Currently, we do not fully understand such differences; one possibility is that men and women researchers essentially contribute equally, but are not rewarded equitably by grants, author roles or tenured positions. Alternatively, the roles of men and women researchers may be different

due to different trajectories during their training, in which case, unequal rewards would be merely a consequence of these different skills and contributions.

The research-hypothesis framework is presented in Figure 1 below. We assessed this research question in a cross-sectional study of original articles published 15 years apart in a single, widely cited US-based medical journal that provides a constant and standard format for reporting author contributions, in contrast to other journals.[13] Our results highlight that research roles are not distributed equally between women and men researchers and that these differences have remained unchanged over a 15-year period. This may be due to justifiable reasons as explained above. However, it is also possible that the academic research milieu perpetuates sexist attitudes and unequal treatment of researchers based solely on their gender. Before we launched ATHENA, we conducted an ancillary study where we explored women's visibility at three important authorship positions (first, last, and corresponding) during the Covid-19 pandemic among 11 biomedical journals from the BMJ Publishing Group.[14] We showed that gender disparities were accentuated for Covid-19 related manuscripts early in the pandemic. These gender disparities in submitted manuscripts narrowed in the most recent pandemic period (Feb-May 2021) compared with the early pandemic (Jan-May 2020). In a second ancillary study, we confirmed the existence of gender and geographical biases during the peer reviewing process which widened during the pandemic.[18]

While we have confirmed others' results[13] that a gender gap may exist at various points during the research process, exploration of gender bias at the point of manuscript evaluation by editors of academic journals is warranted. In a preliminary analysis of all original articles submitted between July and December 2017 to the journal *Clinical Microbiology and Infection*, we found—paradoxically—that women first authors, though fewer in number, had significantly higher acceptance for publication than their male counterparts (45% versus 27%, p=0.003); the editor's gender had no effect on rates of acceptance.[21] This unexpected result obtained in a single journal specializing in infectious diseases and clinical microbiology from original articles submitted over a six-month period is unlikely to be generalizable. Furthermore, gender bias should be assessed in multivariate analyses, independently of other study attributes known to be associated with chances of publication. It has, however, heightened our motivation to test the research hypothesis on a larger sample of journals from various medical domains.

Access to such data is difficult to obtain. The BMJ Publishing Group has agreed to participate and we have received the data of all submissions between January 1, 2012 and May 31, 2021 from 11 of its journals. Four additional journals have also agreed to participate in the ATHENA project (*Clinical Microbiology & Infection*, *Infection Control and Hospital Epidemiology*, *British Journal of Anesthesiology*, *Journal of Antimicrobial Chemotherapy*); three of these have already sent 120 pdfs (60 manuscripts accepted for publication and 60 manuscripts rejected after review) as a pilot study for assessing feasibility. The *Lancet Infectious Diseases* agreed to participate but would not provide pdfs of the manuscripts; they sent all the data initially defined in the protocol from 60 manuscripts accepted for publication and 60 manuscripts rejected after review. All journals that we contacted are presented in Appendix 2 and those who agreed to participate have sent letters of support (Appendices 3 to 6). So we have carefully and successfully woven together a network of journals representing a range of specialties and impact factors whose editors are willing to explore with us the possibility of gender and other biases in their editorial decision-making processes in order to define the problem, if any, and thereafter to identify strategies to solve it.

Figure 1. Framework of possible mechanisms explaining gender bias in the authorship of scientific publications and academic promotion (adapted from reference[13]).

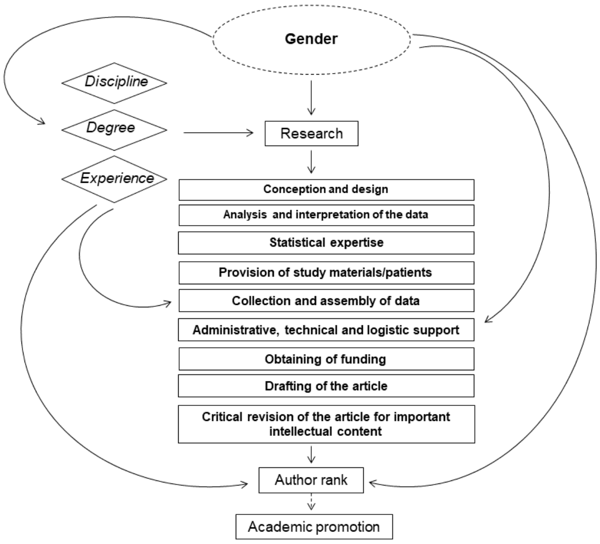

2.3. Detailed research plan

2.3.1. Study rationale

As stated before, while a gender gap is clearly present in the conduct and publication of medical research, no one has yet specifically explored whether that gap could at least partially be due to a bias in journal editors' decisions to accept or decline a submitted manuscript. We propose here to assess the existence of gender bias throughout the editorial decision-making of several medical journals.

2.3.2. Aim and hypothesis

We postulate that acceptance rates for publication should differ by a factor 0.85 between women and men first authors among leading medical journals to confirm a gender bias. We aim to identify the influence of first-author gender on acceptance rates among original research articles sent for peer review by leading medical journals over an eight-year period.

2.3.3. Objectives

Our **primary objective** is to evaluate the influence of first-author gender on acceptance rates of original articles submitted to leading medical journals that were sent for peer review.

Our **secondary objectives** are: 1) to study the influence of corresponding-author gender and last-author gender on acceptance for publication; 2) to test whether the editor's or associate editor's (see glossary) gender modifies the influence of author gender on acceptance for publication; 3) to determine whether the influence of author gender on acceptance for publication varies over an eight-year period; 4) to determine the proportion of women first authors at each stage of the editorial decision-making process; 5) to possibly explore whether the reasons for manuscript rejection differ between women and men first, last and corresponding authors.

2.3.4. Study design

We will conduct a case-control study (main study design) nested in a retrospective cohort of original articles submitted to leading medical journals of various specialties during an eight-year period (January 2012 through December 2019). The cases will be a randomly selected sample of original research articles sent for peer review. Due to low acceptance rates in some of the selected journals, we will conduct a stratified case-control study on the journal.

2.3.5. Study population and eligibility criteria

2.3.5.1. Retrospective cohort study

The study population is all articles submitted for publication in a list of invited medical journals in various medical specialties and with various impact factors or SCImago Journal Rank indicator, between 1 January 2012 and 31 December 2019 (Appendix 2). Medical journals publishing only non-human or basic-science research, case series or review articles will not be invited to participate.

2.3.5.2. Case-control study

The study population will be original research articles and systematic reviews submitted to participating journals that were externally peer reviewed and which focus on clinical and/or translational research. Original articles presenting results of non-human or basic-science research (animals, *in vitro*) will be excluded, as will non-research articles (clinical review articles, editorials, commentaries, protocols and letters). All articles where we are unable to identify the gender of the first author will be excluded.

2.3.6. Selection of medical journals

As the number of medical journals is constantly increasing, we needed to select journals on valuable indicators. Traditionally, journal-based metrics such as Journal Impact Factor (JIF,see glossary) were used to compare scientific output of individuals and institutions. However, this tool has several limitations, among them field specificity and lack of generalizability, and can be subject to manipulations by editorial policy. Indeed, the use of JIF is no longer recommended (San Francisco Declaration on Research Assessment [DORA]).[21] We thus selected leading journals based on the SCImago Journal Rank indicator (see glossary), inviting those journals ranked in the first and second quartile to participate. We also invited journals based on opportunities. Currently, 27 of the 41 invited journals have agreed to participate (see Appendix 3-6, letters of support), and 11 declined (see Appendix 2). Among, the 27 journals who agreed to participate, four journals provided limited access to their databases which did not allow us to apply our definitions and selection for cases and controls so we have had to exclude them. Finally, we only selected journals with a periodicity of publication of at least every two months and journals that receive over 100 research article submissions a year (Appendix 2). The application of these eligibility criteria left 23 journals willing to participate and share data.

2.3.7. Outcome measures and data collection

2.3.7.1. Outcomes

In the case-control study, cases (primary outcome) will be **articles accepted for publication after peer review and** controls will be **articles rejected for publication after peer review**.

In the retrospective cohort study, **the acceptance rate of all submitted articles** by gender of first, last and corresponding author (secondary outcomes), will be determined. All original research articles and systematic reviews will be included. Additionally, we will record the rates of "reject without peer review", revision requests, and later rejection throughout the manuscript's cycle.

2.3.7.2. Exposure variables and other confounders

The main exposure factor of interest is **first-author gender**. Other exposure factors assessed are: corresponding and last-author gender, handling-editor gender.

We will also collect data on: year of submission, study design, sample size, statistical significance of main results, funding disclosure, first author's country of affiliation (reflecting somehow the author's ethnicity/race), number of authors on the byline, number of reviewers who peer reviewed the manuscript, number (proportion) of women among all reviewers, number (proportion) of reviewers with same region of affiliation as the first author, and first author's degrees (MD, PhD). If available, we will extract the reasons for rejection mentioned in the journal decision letter.

2.3.8. Random selection of original articles in case-control study

Random selection of original articles will be stratified by journal. Once journals agree to participate, we will ask for a list of all manuscripts (identified by their journal tracking number) submitted between 1 January 2012 and 31 December 2019. We will request additional information on whether the articles were peer reviewed and the final editorial decision: "accepted for publication" or "rejected". We will randomly order the list of accepted and rejected manuscripts after peer review. In total, we calculated that among the 23 participating journals, depending on the first journal edition, we will have a total of 172 journal-years (BMJ Global Health and BMJ Paediatrics Open started respectively in January 1, 2016 and January 1, 2017). Thus based on our sample size estimation (see section 2.3.9.1) we will include 20 cases and 20 journals per journal-year, corresponding to a maximum of 160 cases and 160 controls per journal. In a second step, PDFs of all selected articles will be retrieved to extract additional data on study attributes.

2.3.8.1. Data extraction

2.3.8.1.1. Extraction for the case-control study

We will complete data extraction (study design, statistical significance of main result, etc.) from the manuscripts by using text mining techniques. For this specific task, we will collaborate with the Swiss Institute of Bioinformatics (SIB), Bibliomics and Text Mining Group (BiTeM, Patrick Ruch) through a study partnership. All prespecified variables will be automatically extracted using an algorithm created by our project partner at the SIB and recorded in a database. Data on manuscripts, authors, editors and reviewers involved will be obtained directly from the journals and merged with the dataset (list of variables; see appendix 7).

2.3.8.1.2. Extraction for the retrospective cohort study

Regarding variables for the retrospective cohort, most variables will be directly exported from the journal systems in plain text (CSV or XLS). These variables will include the date of submission, the article's first decision and last decision, the authors' the countries of affiliation, and the journal identifier (case report form; see appendix 8). We will complete data extraction (for study design, statistical significance of main result, etc.) from the abstracts of the manuscripts by using text mining techniques elaborated by our project partner.

2.3.8.1.3. Identification of authors' gender

The identification of first, corresponding and last author's gender will be performed independently of the editorial decision (accepted or rejected for publication) via an internet search using specific tools by a collaborator in the research team. This will be performed separately for the case-control study and for the retrospective cohort study. Determination of authors' gender will be based on first name, middle name, salutation and the country of the author's affiliation. We will use binary gender identification that will not be based on the self-reported gender identity. We will determine gender by using a sequential four step procedure.[14] Firstly, we will use both first name and country to search in the Gender API (https://gender-api.com/en) website. Gender API provides gender determination with an accuracy probability from 50% to 100% (under 50% an unknown status is attributed), gives the number of samples retrieved to search for the information, ensures that the level of misclassification is around 5%, and has the highest coverage on the variability in name origins.[23] We will set gender accuracy above 60% as the minimum criterion for inclusion in the primary gender determination. Secondly, for all authors with undetermined or unknown gender based on the above, we will use the authors' given middle names and country to search in Gender API. Thirdly, we will use the online service genderize.io (http://genderize.io) to determine gender on the basis of first names and middle names, also using an accuracy above 80% as the minimum criterion. Fourthly, for still undetermined gender, we will use the authors' title/salutation and attribute it male gender to "Mr" or "M" and female gender to "Miss," "Mrs," and "Ms." Where none of these methods determine the gender, we will record the gender as undetermined.

2.3.8.1.4. Final dataset for case-control study

We will disaggregate the editorial decision variables from the submission data so that determination of author's gender and all other data extraction will be done blinded to editorial decision. Finally, for analysis we will combine the separate databases using the manuscript identity number.

2.3.9. Statistical methods

2.3.9.1. Sample size

As described above, we will consider an odds ratio of 0.85 for the acceptance for publication by first-author gender (0.85 for women versus men), to indicate the presence of gender bias. We assume a proportion of women first authors of 40% among cases and 44% among controls using OR=0.85.[11] Taking into account a type-1 error at 5% (two-sided) with a study power of 90% and a correlation coefficient for exposure between matched controls and cases at 0.03 (to take into account some clustering at the journal level), this would require 2800 articles accepted and 2800 articles rejected after peer review. To take into account at least 20% of missing values among modelled variables,[19] we will select a total of 3500 accepted and 3500 rejected manuscripts. We will randomly select an

equal number of articles per journal-year (20 cases and 20 controls) corresponding to a maximum of 160 cases and 160 controls for each journal. Currently, 23 journals have agreed to participate.

2.3.9.2. Statistical analyses

Statistical analyses will be done by the postdoctorate under the supervision of AGA. We will present descriptive statistics for the proportions of women first authors that were: rejected without external review; sent for external peer review; rejected, accepted or invited to revise a manuscript after external peer review; and rejected and accepted after revision. To take into account clustering at the journal level, we will perform mixed-effects logistic regression to model the influence of the first author's gender on the odds of acceptance for publication, after adjustment for the confounders and covariates (cf. appendix 7) and using the journal as a random effect. We will perform the same logistic regression model to answer our secondary objectives to study the influence of corresponding-author gender or last-author gender on the acceptance for publication. To test whether the handling editor's gender or reviewers' gender modifies the association between first-author gender and the acceptance rate, we will include an interaction term between these two predictors. We will also test interactions of first author gender, respectively with the scope of the journal and with time on a yearly scale. Journals will be anonymised, therefore separate journal effects will not be presented when we publish the results but we will provide journal level data to individual journals upon request.

In the retrospective cohort, we will describe and compare, using Chi-2 tests, the proportion of women first authors for articles at the following points in the decision-making process: rejection without review; sent for peer review; rejected, accepted and sent for revision after peer review; and between articles rejected at any stage versus articles accepted for publication. To test whether the reasons for rejection mentioned in decision letters varies between men and women first (then corresponding and last) authors, we will include an interaction term between these two predictors in a mixed-effects logistic regression model with rejection as the dependent variable and journal as the random factor. All analyses will be performed using STATA 17.0 IC (StataCorp LP, College Station, TX, USA) and R software (version 4.0.5).

2.3.10. Ethical considerations

As the project will not involve human participants but data routinely collected from articles submitted for publication in various medical journals, we do not expect any major ethical issues. However, to guarantee the anonymity of the authors, reviewers, and editors, we will remove personal identifying variables from the final database, all names of individuals will be removed and only their gender included. We will also preserve journal anonymity by presenting results without naming the journals; journals will be identified by a coded variable. The protocol was submitted to the Geneva Cantonal Ethics Commission on March 23, 2019. It was exempted from ethical approval on April 24, 2019 but received full support (appendix 1). Personal data will be provided by journals under confidentiality agreements or research contracts.

2.4. Schedule and milestones

2.4.1. Timeframe

**Tasks (*person in charge*)**	**2021**												**2022**											
	**1**	**2**	**3**	**4**	**5**	**6**	**7**	**8**	**9**	**10**	**11**	**12**	**1**	**2**	**3**	**4**	**5**	**6**	**7**	**8**	**9**	**10**	**11**	**12**
Confidentiality agreement procedure from journals																								
Procedure for gender determination																								
Exploration of available data in the BMJ database (*postDoc*)																								
Journals recruitment (*main applicant and postdoc*)																								
Creation of algorithms for automatic data extraction in pdfs (*Swiss Institute of Bioinformatics, SIB*)																								
Data acquisition for the case-control study in the pdfs (*SIB*)																								
Data acquisition for the case-control study in the journal databases (*journal editors, main applicant and postDoc*)																								
Collection of information on the editorial decision-making process at each participating journal (*main and co-applicant*)																								
Collection of data for retrospective cohort study (*postDoc*)																								
Data analysis and interpretation of the case-control study (*postDoc, main applicant*)																								
Writing of main manuscript (case-control study), conferences (*postDoc, main/co applicants, partners*)																								

**Tasks (*person in charge*)**	**2023**					
	**1**	**2**	**3**	**4**	**5**	**6**
Writing/critical revision of main manuscript (case-control study) (*postDoc, main/co applicants, partners*)	■	■				
Dissemination of study results findings (*main/co-applicants, postDoc*)			■	■	■	■
Data analysis and interpretation of secondary study (*postDoc, main/co applicants*)			■	■	■	■
Writing/critical revision of secondary manuscript (case-control study), conferences (*postDoc, main/co applicants, partners*)		■	■	■		
Dissemination of second study results findings (*main/co-applicants, postDoc*)					■	■

2.5. Relevance and impact

2.5.1. Scientific relevance

Before it can reach publication, an original article is left in the hands of individuals in the editorial team who could, as in society, apply their own rules and possibly gender discrimination, whether consciously or not. All submitted manuscripts go through a preliminary editorial screening during which assessments are made as to whether the topic is within the journal's scope, author instructions were respected, and general quality standards are met; it is then sent out for peer review; once the required number of reviews is received, the editor(s) consider the experts' opinions and make an initial decision to accept, reject or request revision, or take it to a committee and/or statistical reviewer for further assessment. Revised papers are re-evaluated by the original handling editor, who either makes a decision (or refers it back to a committee that may include a statistical advisor) or sends the manuscript for further peer review prior to making a decision. Editors may request multiple manuscript revisions. This editorial decision-making process may vary in the assessment criteria used, depending on the journals; the final decision could be left to a single individual (the handling editor) or to a group of persons (Figure 2). The BMJ, one of the largest participating journals, includes patient and public reviewers in the review process (Figure 3).

This research will provide important insights into the mechanism of gender bias in medical research. We will specifically explore whether gender might interfere with chances of article publication. We are looking for additional reasons to explain significant differences in the scientific productivity (lower number of publications and smaller h-index in women compared to men) between women and men researchers.[10,19,24,25] Gender might not be the only factor inducing bias in the assessment of research productivity; we would need to acknowledge intersectionality, especially with regards to race and ethnicity of the authors, and the risk of additional and compounded bias. For example, an editor/reviewer could vaguely assume someone's race or ethnicity based on their first name, and conscious/unconscious bias could affect their decision making on top of gender bias. It is less likely that other aspects of diversity, e.g. age or sexual orientation, could creep into the editorial and peer review process as it is almost impossible to guess these personal characteristics based on the first name of a person without knowing them. This could potentially be a limitation as up until now, journals do not collect or report on the race/ethnicity of authors. However, we will use a proxy by describing the authors' country of affiliation. Research results will be published in an open-access journal and presented at a gender-centred conference (*e.g.*, Women in Science and Healthcare symposium, Women in STEM conference).

Figure 2. Editorial decision-making process.

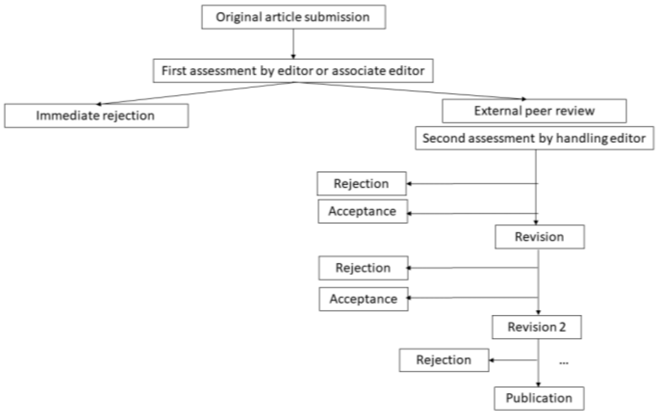

Figure 3. Editorial & review decision-making process at the BMJ Group.

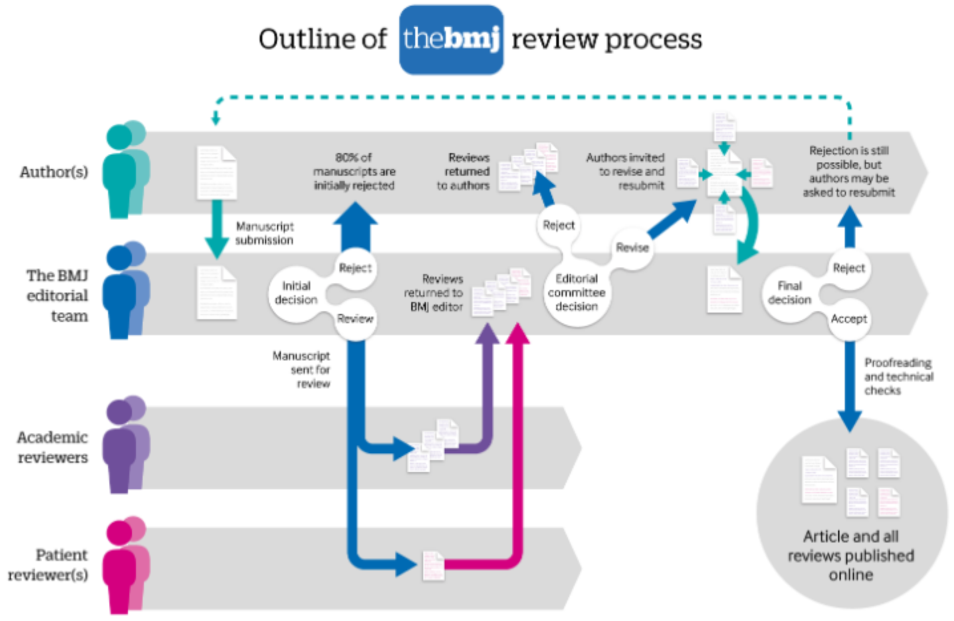

2.5.2. Broader impact

The identification of a gender bias at the point of editorial decision-making, would add to the growing evidence that the slower progression of women in academia is explained by a sequence of various barriers throughout the career path (e.g., selective choice in career progression due to career interruptions mainly explained by maternity leaves and parenthood, difficulties in both conducting clinical and research activities). It would identify an important target for future strategies aimed at reducing discrimination. Indeed, repeated, insidious acts of gender discrimination cumulatively create inequities that disadvantage women in their likelihood of achieving leadership positions. Without specific targets and strategies, the changing of mentalities and societal biases requires a good deal of time. On the contrary, pragmatic measures for specific objectives could be rapidly constructed and introduced into the editorial policies of medical journals. Currently, some editors already implement some measures to involve more women in the editorial and review process, such as deliberately inviting more women peer reviewers, having more women as editorial board members, etc.[26] If we not observe any gender bias at this stage of the academic pipeline, this will be valuable information for other researchers, who may then work to shift focus and resources to other, upstream points along the pipeline.

3. Bibliography

1. Moberly, T. Number of women entering medical school rises after decade of decline. *BMJ* **360**, (2018).
2. Hostettler, S. & Kraft, E. Statistique médicale 2018 de la FMH: peu de femmes aux postes de cadre. *Bull. Médecins Suisses* **100**, 411–416 (2019).
3. Dubach, P., Graf, I. & Stutz, H. Dual-career couples at Swiss Universities: Gender Campus. (2013).
4. Université de Genève. *Étudiantes et étudiants, diplômes et personnel*. (2016).
5. Smith, V., Bethune, C. & Hurley, K. F. Examining Medical Student Specialty Choice Through a Gender Lens: An Orientational Qualitative Study. *Teach. Learn. Med.* **30**, 33–44 (2018).
6. Heiligers, P. Gender differences in medical students' motives and career choice. *BMC medical education* (2012). doi:10.1186/1472-6920-12-82
7. Lefevre, J. H., Roupret, M., Kerneis, S. & Karila, L. Career choices of medical students: a national survey of 1780 students. *Med. Educ.* **44**, 603–612 (2010).
8. Sambunjak, D., Straus, S. & Marusić, A. Mentoring in academic medicine: a systematic review. *JAMA* (2006). doi:10.1001/jama.296.9.1103
9. Witteman, H. O., Hendricks, M., Straus, S. & Tannenbaum, C. Are gender gaps due to evaluations of the applicant or the science? A natural experiment at a national funding agency. *The Lancet* **393**, 531–540 (2019).
10. Moss-Racusin, C. A., Dovidio, J. F., Brescoll, V. L., Graham, M. J. & Handelsman, J. Science faculty's subtle gender biases favor male students. *Proc. Natl. Acad. Sci. U. S. A.* **109**, 16474–16479 (2012).
11. Filardo, G. *et al.* Trends and comparison of female first authorship in high impact medical journals: observational study (1994-2014). *BMJ* **352**, (2016).
12. Bendels, M. H. K., Müller, R., Brueggmann, D. & Groneberg, D. A. Gender disparities in high-quality research revealed by Nature Index journals. *PLOS ONE* **13**, e0189136 (2018).
13. Gayet-Ageron, A., Poncet, A. & Perneger, T. Comparison of the contributions of female and male authors to medical research in 2000 and 2015: a cross-sectional study. *BMJ Open* **9**, e024436 (2019).
14. Gayet-Ageron, A., Ben Messaoud, K., Richards, M., Schroter, S. Female authorship of covid-19 research in manuscripts submitted to 11 biomedical journals: cross sectional study. *BMJ* **375**, n2288 (2021).
15. Edwards, H. A., Schroeder, J. & Dugdale, H. L. Gender differences in authorships are not associated with publication bias in an evolutionary journal. *PLOS ONE* **13**, e0201725 (2018).
16. Fox, C. W., Burns, C. S. & Meyer, J. A. Editor and reviewer gender influence the peer review process but not peer review outcomes at an ecology journal. *Funct. Ecol.* **30**, 140–153 (2016).
17. Fox, C. W. & Paine, C. E. T. Gender differences in peer review outcomes and manuscript impact at six journals of ecology and evolution. *Ecol. Evol.* **9**, 3599–3619 (2019).
18. Ben Messaoud, K., Richards, M., Schroter, S., Gayet-Ageron, A. Analysis of peer reviewers' response to invitations by gender and geographical region: cohort study of manuscripts reviewed at 21 biomedical journals before and during covid-19 pandemic. *BMJ* **381**, e075719 (2023).
19. Macaluso, B., Larivière, V., Sugimoto, T. & Sugimoto, C. R. Is Science Built on the Shoulders of Women? A Study of Gender Differences in Contributorship. *Acad. Med.* **91**, 1136–1142 (2016).

20. Lee, K. P., Boyd, E. A., Holroyd-Leduc, J. M., Bacchetti, P. & Bero, L. A. Predictors of publication: characteristics of submitted manuscripts associated with acceptance at major biomedical journals. *Med. J. Aust.* **184**, 621–626 (2006).
21. Huttner, A. & Friedman, J. Author gender and editors' decisions at Clinical Microbiology and Infection. *Clinical microbiology and infection : the official publication of the European Society of Clinical Microbiology and Infectious Diseases* (2019). doi:10.1016/j.cmi.2019.01.012
22. Way, M. & Ahmad, S. A. The San Francisco Declaration on Research Assessment. *J. Cell Sci.* **126**, 1903–1904 (2013).
23. Santamaría, L. & Mihaljević, H. Comparison and benchmark of name-to-gender inference services. *PeerJ Comput. Sci.* **4**, e156 (2018).
24. Myers, S. P. *et al.* A Systematic Review of Gender-Based Differences in Hirsch Index Among Academic Surgeons. *J. Surg. Res.* **236**, 22–29 (2019).
25. Diamond, S. J. *et al.* Gender Differences in Publication Productivity, Academic Rank, and Career Duration Among U.S. Academic Gastroenterology Faculty. *Acad. Med.* **91**, 1158–1163 (2016).
26. Clark, J. & Horton, R. What is The Lancet doing about gender and diversity? *The Lancet* **393**, 508–510 (2019).

Glossary.

- **Associate editor**: a journal editor who may be assisted by one or more associate editors to manage schedules and coordinate workflow. Responsibilities vary depending on the specific position, but they may include reviewing and editing the work of staff or freelance writers, enforcing deadlines, and overseeing the production details of one or more publications, such as newspapers, magazines, books and/or websites.

- **Journal Impact Factor (JIF)**: The impact factor is a measure of the frequency with which the "average article" in a journal has been cited in a particular year or period. The annual JCR impact factor is a ratio between citations and recent citable items published. Thus, the impact factor of a journal is calculated by dividing the number of current year citations by the source items published in that journal during the previous two years.

- **SCImago Journal Rank** (SJR indicator): is a measure of scientific influence of scholarly journals that accounts for both the number of citations received by a journal and the importance or prestige of the journals where such citations come from. A journal's SJR is a numeric value indicating the average number of weighted citations received during a selected year per document published in that journal during the previous three years. Higher SJR values are meant to indicate greater journal prestige.

Appendix 1. Decision of the local ethical committee.

REPUBLIQUE ET CANTON DE GENEVE
Département de la sécurité, de l'emploi et de la santé (DSES)

Direction générale de la santé

Service du pharmacien cantonal
Commission cantonale d'éthique de la recherche (CCER)
Rue Adrien-Lachenal 8
1207 Genève

Dre Angèle Gayet-Ageron
Service d'Epidémiologie Clinique
Hôpitaux Universitaires de Genève
Rue Gabrielle Perret-Gentil 4
1211 Geneva 14

N/réf. : OH/md/fr

Genève, le 24 avril 2019

Décision de la Commission cantonale d'éthique de la recherche
CCER du 16 avril 2019

**No de projet**	2019-00540
**Titre du projet**	Assessment of gender bias in editorial decision-making: does author gender have an effect on the manuscript acceptance? An observational study of leading medical journals
**Investigateur principal**	Dr Angèle Gayet-Ageron
**Promoteur**	Dr Angèle Gayet-Ageron
**Centres**	Dr Angèle Gayet-Ageron, University hospitals of Geneva; University of Geneva, Faculty of Medicine, Geneva

Procédure de décision

☐ Procédure ordinaire ☒ Procédure simplifiée ☐ Procédure présidentielle

Décision

Dr Angèle Gayet-Ageron, University hospitals of Geneva; University of Geneva, Faculty of Medicine, Geneva

☒ Non entrée en matière

Après avoir soigneusement étudié les documents fournis, la CCER déclare que cette étude ne rentre pas dans le champ d'application des lois qui régissent la recherche sur l'être humain en Suisse (LRH, ORH et OClin), et ne nécessite donc pas d'autorisation formelle de sa part. Néanmoins, la CCER salue l'intérêt de ce projet et en soutient fortement la réalisation.

Signature

Dr Olivier Huber, CC

Appendix 2. Journals contacted and which have accepted to participate or declined the invitation.

Journals	2018 SJR[a] (quartile)	Participation		Selection		Peer review model
		Accepted	Declined	Yes	No	
American Journal of Transplantation	3.200 (Q1)		X			
Annals of the Rheumatic Diseases(BMJ)	7.081 (Q1)	X		X		Single anonymised
Antimicrobial Resistance & Infection Control[2]	1.681 (Q1)		X			
Archives of Disease in Childhood (BMJ)	1.303 (Q1)	X		X		Single anonymised
British Journal of Ophtalmology (BMJ)	1.872 (Q1)	X		X		Single anonymised
The BMJ	2.881 (Q1)	X		X		Open and published
BMJ Evidence-Based Medicine	0.56 (Q2)	X			X	Single anonymised
BMJ Global Health	2.111 (Q1)[b]	X		X		Single anonymised
BMJ Health & Care Informatics	0.427 (Q2)[b]	X			X	Single anonymised
BMJ Open	1.321 (Q1)	X		X		Open and published
BMJ Open Quality	0.436 (Q2)[b]	X			X	Single anonymised
BMJ Quality and Safety	2.833 (Q1)	X		X		Triple anonymised
BMJ Paediatrics Open	0.82 (Q1)[b]	X		X		Open peer review
British Journal of Sports Medicine (BMJ)	4.141 (Q1)	X		X		Single anonymised
Clinical Infectious Diseases	4.396 (Q1)		X			
Clinical Microbiology and Infection	2.651 (Q1)	X[c]		X		
ESMO Open	2.409 (Q1)[b]		X			Single anonymised
European Journal of Anaesthesiology	1.287 (Q1)	X[c]			X	
Eurosurveillance	3.876 (Q1)		X			
Gut	7.085 (Q1)	X		X		Single anonymised
Heart	2.635 (Q1)	X		X		Single anonymised
Infection Control and Hospital Epidemiology	1.540 (Q1)	X[c]			X	
Journal of Antimicrobial Chemotherapy	2.139 (Q1)	X		X		
Journal of Epidemiology and Community Health (BMJ)	2.028 (Q1)	X		X		Single anonymised
Journal of Hospital Infection	1.474 (Q1)	X			X	
Journal of Medical Genetics(BMJ)	3.017 (Q1)	X		X		Single anonymised
Journal of Neurology, Neurosurgery, and Psychiatry	3.21 (Q1)	X		X		Single anonymised
Journal of NeuroInterventional Surgery (BMJ)	1.613 (Q1)	X		X		Double anonymised
Journal of Clinical Pathology (BMJ)	1.085 (Q1)	X		X		Single anonymised
JAMA	7.477 (Q1)		X			
Lancet Infectious Diseases	9.462 (Q1)	X			X	
Lancet Oncology	18.073 (Q1)		X			
Lancet Neurology	12.285 (Q1)		X			
Lancet	15.871 (Q1)		X			
Occupational and Environmental Medicine	1.671 (Q1)	X		X		Single anonymised
PLoS One	1.100 (Q1)		X			
PLoS Medecine	6.626 (Q1)		X			

Postgraduate Medical Journal	0.69 (Q2)	X		X		Single anonymised
Sexually Transmitted Infections	1.672 (Q1)	X		X		Single anonymised
Thorax	3.697 (Q1)	X		X		Single anonymised
Tobacco Control	3.218 (Q1)	X		X		Single anonymised

[a]SJR: SCImago Journal Rank indicator; [b]2020 SJR, [c]full-texts sent

Appendix 3. Letters of support to the project from the BMJ Group.

Dr Angèle Gayet-Ageron
Médecin Adjointe Agrégée
Service d'Epidémiologie Clinique
Responsable de l'Unité d'Appui Méthodologique du CRC
Hôpitaux Universitaires de Genève et Faculté de Médecine
Université de Genève
Rue Gabrielle Perret-Gentil 4
CH 1211 Genève 14

16 September 2019

Dear Drs. Angèle Gayet-Ageron and Angela Huttner,

Re: Assessment of gender bias in editorial decision-making: does author gender have an effect on the manuscript acceptance? An observational study of leading medical journals

In my capacity as senior researcher at BMJ, I fully support Drs. Angèle Gayet-Ageron (main applicant) and Angela Huttner, (co-applicant) in this endeavor to explore the existence of gender bias in editorial decision-making among manuscripts submitted to medical journals.

We are delighted that you have invited BMJ Publishing Group to work with you on this interesting and important project. We publish more than 60 journals and so far 16 journals, including *The BMJ* and some of our other high impact clinical journals, have expressed a keen interest to be included in your research. The proposed work is highly relevant and in line with the research focus of the journals at BMJ Group. We will provide access to all the data needed including permission to access our electronic manuscript tracking systems. As you know the BMJ has its own programme of research and regularly collaborates with external researchers so we have a process for providing you with the data you need.

Yours sincerely

Sara Schroter, PhD
Senior Researcher, BMJ

BMA House
Tavistock Square
London WC1H 9JR
United Kingdom

T : +44 (0)20 7387 4410
W: bmj.com

BMJ Publishing Group Ltd
Registered office:
BMA House
Tavistock Square
London WC1H 9JR
United Kingdom

Registered in England No. 3102371
VAT registered No. GB 674 7384 91

Appendix 4. Letters of support to the project from the Clinical Microbiology & Infection.

CMI CLINICAL MICROBIOLOGY AND INFECTION

ESCMID

Professor Leonard Leibovici
Editor-in-Chief
email: leibovic@post.tau.ac.il

ESCMID EUROPEAN SOCIETY OF CLINICAL MICROBIOLOGY AND INFECTIOUS DISEASES

17 September 2019

To Whom It May Concern,

In my capacity as Editor-in-Chief of *Clinical Microbiology and Infection* I fully support Drs. Angèle Gayet-Ageron and Angela Huttner in this endeavor to explore the existence of a gender bias in editorial decision-making vis-à-vis the original article first author in leading medical journals.

The proposed work is highly relevant and well in line with the research focus of the journal. Constructive discussions and important contact with 23 journals from the BMJ Group have already been made, and I can guarantee availability of the necessary data from the CMI.

Sincerely,

Professor Leonard Leibovici
Editor-in-Chief
Clinical Microbiology and Infection

Julia Friedman
Editorial Manager
Clinical Microbiology and Infection

CMI Editorial Office
Beilinson Hospital, Rabin Medical Center, Department of Medicine E
Derech Ze'ev Jabotinsky 39, Petah Tikva 4941492 Israel

Appendix 5. Letters of support to the project from the Journal of Antimicrobial Chemotherapy.

Journal of Antimicrobial Chemotherapy
The Journal of the British Society for Antimicrobial Chemotherapy

Editor-in-Chief:
Dr Peter Donnelly

Dr. Med. Angèle GAYET-AGERON and Dr. Angela Huttner
University Hospitals of Geneva
4, rue Gabrielle-Perret-Gentil
CH-1211 Genève 1
Switzerland

17th September 2019

Dear Drs Gayet-Ageron and Huttner,

Re: “First-author Gender study”: Assessment of gender bias in editorial decision-making: does first author gender have an effect on manuscript acceptance? An observational study of medical journals.

Thank you for the invitation to join this project ““First-author Gender study”: Assessment of gender bias in editorial decision-making: does first author gender have an effect on manuscript acceptance? An observational study of medical journals”

Having discussed this with the British Society of Antimicrobial Chemotherapy, Oxford University Press and our editorial team, I would like to confirm the participation of the Journal of Antimicrobial Chemotherapy in this project. I am also happy to lend my support as a partner to this timely investigation. We are often accused of various forms of bias including that relate to gender so it would be good to have the facts at our disposal, no matter what the results of this study turn out to be, so that we can take whatever steps are necessary to remedy any bias that is identified.

I look forward to this moving forward and will do my utmost to ensuring that the study reaches a successful conclusion.

Yours sincerely,

J Peter Donnelly, BSc PhD FRCPath
Editor in Chief
Journal of Antimicrobial Chemotherapy

De Hoefkamp 1096
6545 MD Nijmegen
The Netherlands
Tel: +31 (0)24 663 5188
email: p.donnelly@usa.net

JAC Editorial Office
Griffin House, 53 Regent Place
Birmingham B1 3NJ
United Kingdom

Tel: +44 121 262 1830
Email: jac@bsac.org.uk
Website: http://jac.manuscriptcentral.com

British Society for Antimicrobial Chemotherapy
Registered Charity 1093118
Registered as a company limited by guarantee in England & Wales No. 4443910

Appendix 6. Letters of support to the project from the Lancet Infectious Diseases.

THE LANCET Infectious Diseases

September 23rd, 2019

To whom it may concern,

In our capacity as Editor-in-Chief of the *Lancet Infectious Diseases* journal and Senior Editor of *The Lancet Infectious Diseases*, we fully support Drs Angèle Gayet-Ageron, main applicant and Angela Huttner, co-applicant in this endeavour to explore the existence of a gender bias in editorial decision-making among manuscripts submitted to medical journals. We will participate in data collection regarding our journal.

The proposed work is highly important and fits with the Lancet values and our commitment to increase gender equity, diversity, and inclusion in research and publishing.

With our best wishes,

The Lancet Infectious Diseases
125 London Wall
London EC2Y 5AS
United Kingdom

t +44 (0)20 7424 4910
f +44 (0)20 7424 4915
www.thelancet.com

John McConnell
Editor
The Lancet Infectious Diseases
125 London Wall
London EC2Y 5AS
UNITED KINGDOM

Marco De Ambrogi, PhD,
Senior Editor,
The Lancet Infectious Diseases
Elsevier
125 London Wall
London EC2Y5AS
UNITED KINGDOM

The Lancet, Elsevier Inc.
360 Park Avenue South
New York, NY 10010, USA.
Registered office: The Boulevard, Langford Lane, Kidlington, OX5 1GB. Registered in England Reg No. 730 6955 26

Appendix 7. Data extracted for the case-control study.

**Variables**	**Description**
Unique ID of manuscript	tracking number generated during submission process
Journal identifier	##
Scope of the journal	1= infectious diseases journal 2= family medicine 3=internal medicine 4=others
Date of submission	__ / __ /____
Handling editor’s gender	0=man, 1=woman
Study design	1=randomised-controlled trial, 2= cohort, prospective, 3=cohort retrospective, 4=cross-sectional, 5=case-control, 6=case reports or case series, 7=systematic review without meta-analysis, 8=systematic review and meta-analysis, 9= other
Effective sample size	####
Statistical significance of main results (p<0.05)	0=no, 1=yes, 8=not applicable 9= unknown
Funding source	0=no funding, 1=public funding, 2=private/industry, 3=mixed funding
Funding disclosure	0=no, 1=yes, 2=not mentioned
Study country(ies) of affiliation	[...]
Multi center study	0=no, 1=yes
Number of participating centers	###
Number of authors on the byline	##
Degrees for the first author	1=MD, 2=PhD, 3=MSc or equivalent, 4=RN, 5=mixed PhD & MD...
First author’s gender	0=man, 1=woman
Corresponding author’s gender	0=man, 1=woman
Last author’s gender	0=man, 1=woman
Funding source	1=private, 2= public, 3=NGO, 4=mixed
Reviewers’ degrees	1=MD, 2=PhD, 3=MSc or equivalent, 4=RN, 5=mixed PhD & MD...
Number of submissions	#
Number of revisions	#
Number of reviewers	#
Number (proportion) of women reviewers	#
Number of reviewers from same region as first author	#

Appendix 8. Case-report form for the retrospective cohort study.

**Variables**	**Description**
Unique ID of manuscript	tracking number generated during submission process
Journal identifier	##
Scope of the journal	1= general medical journal 2= specialist journal
Date of submission	__ / __ /____
Study design	1=randomised-controlled trial, 2= cohort, prospective, 3=cohort retrospective, 4=cross-sectional, 5=case-control, 6=case reports or case series, 7=systematic review without meta-analysis, 8=systematic review and meta-analysis, 9= other
First author's gender	0=man, 1=woman
Corresponding author's gender	0=man, 1=woman
Study country(ies) of affiliation	[...]
Number of authors on the byline	##
Final decision	1=immediate rejection, 2=sent for peer-review, 3=rejected after 1st round of peer-review, 4=rejected after several rounds of peer-review, 5=accepted for publication
Date of final decision	__ / __ /____
Study design	1=randomised-controlled trial, 2= cohort, prospective, 3=cohort retrospective, 4=cross-sectional, 5=case-control, 6=case reports or case series, 7=systematic review without meta-analysis, 8=systematic review and meta-analysis, 9= other
Actual sample size	####
Statistical significance of primary outcome results ($p<0.05$)	0=no, 1=yes, 8=not applicable 9= unknown
Funding source	0=no funding, 1=public funding, 2=private/industry, 3=mixed funding
Funding disclosure	0=no, 1=yes, 2=not mentioned
Study country(ies) of affiliation	[...]
Multi center study	0=no, 1=yes
Number of participating centers	###
Number of authors on the byline	##
First author's degrees	1=MD, 2=PhD, 3=MSc or equivalent, 4=RN, 5=mixed PhD & MD...
Funding disclosure	1=private, 2= public, 3=NGO, 4=mixed
Reviewers' degrees	1=MD, 2=PhD, 3=MSc or equivalent, 4=RN, 5=mixed PhD & MD...
Number of submissions	#
Number of revisions	#
Number of reviewers	#
Number (proportion) of women reviewers	#
Number of reviewers from same region as first author	
